# The Evolution and Diversity of Interleukin-17 Highlight an Expansion in Marine Invertebrates and Its Conserved Role in Mucosal Immunity

**DOI:** 10.3389/fimmu.2021.692997

**Published:** 2021-07-27

**Authors:** Amaro Saco, Magalí Rey-Campos, Umberto Rosani, Beatriz Novoa, Antonio Figueras

**Affiliations:** ^1^Institute of Marine Research (IIM), National Research Council (CSIC), Vigo, Spain; ^2^Department of Biology, University of Padova, Padova, Italy

**Keywords:** interleukin, IL-17, comparative genomics, mucosal immunity, evolution, cytokines, comparative immunology, mussel

## Abstract

The interleukin-17 (IL-17) family consists of proinflammatory cytokines conserved during evolution. A comparative genomics approach was applied to examine IL-17 throughout evolution from poriferans to higher vertebrates. Cnidaria was highlighted as the most ancient diverged phylum, and several evolutionary patterns were revealed. Large expansions of the IL-17 repertoire were observed in marine molluscs and echinoderm species. We further studied this expansion in filter-fed *Mytilus galloprovincialis*, which is a bivalve with a highly effective innate immune system supported by a variable pangenome. We recovered 379 unique IL-17 sequences and 96 receptors from individual genomes that were classified into 23 and 6 isoforms after phylogenetic analyses. Mussel IL-17 isoforms were conserved among individuals and shared between closely related Mytilidae species. Certain isoforms were specifically implicated in the response to a waterborne infection with *Vibrio splendidus* in mussel gills. The involvement of IL-17 in mucosal immune responses could be conserved in higher vertebrates from these ancestral lineages.

## Introduction

Conserved innate immunity mechanisms based on nonself recognition, signal transduction and immune gene activation are essential for effective protection against bacterial and viral invasions. Invertebrate species present a wide range of recognition mechanisms and molecules that specifically bind pathogens (molecules such as lectins, C1q-containing proteins, fibrinogen-like receptors or FREPs, Toll-like receptors, peptidoglycan recognition proteins, *etc.*), as well as effector agents against infection (antimicrobial peptides, lysozymes, proteases and protease inhibitors) (1). Because of the complexity of adaptive systems in higher vertebrates, certain invertebrate species represent a strong model to study the innate immune mechanisms acquired and conserved during evolution, of which great efficiency and importance have been demonstrated.

The signaling pathways induced by cytokines link recognition molecules with immune effectors, so they are essential for understanding how immune mechanisms are finely regulated. The existence of cytokines, which are classified as interleukins, chemokines and interferons in vertebrates, has been slowly revealed in several invertebrate lineages (2, 3). Cytokines are soluble immune-regulatory molecules that can modulate the expression of immune genes in defensive cells and lead to proinflammatory and innate immune signaling functions from mucosal or epithelial cells under infection situations (4, 5).

Interleukin 17 (IL-17) is one of the most conserved cytokines among animal phyla; therefore, it is thought to be a key player in innate immunity. IL-17 was originally identified in humans by Rouvier et al. (6) as a cytolytic T lymphocyte (CTL)-associated antigen, and it was cloned and studied for the first time as an interleukin by Yao et al. (7). In humans, IL-17 is a family of proinflammatory cytokines comprising six members (named IL-17A-F) and five receptors (IL-17RA-E) (8). IL-17 is known to be produced by activated T lymphocytes and other cell types implicated in innate immunity, such as mucosal epithelial cells (4, 9). Their mode of action is based on union into dimers (homodimers or heterodimers) whose activity is dependent on attachment to their receptors (IL-17Rs). Receptors possess a cytoplasmic conserved domain (SEFIR) that interacts with adaptor proteins to induce downstream signal transduction pathways to activate transcription factors such as NF-kB and the expression of immune and proinflammatory target genes such as cytokines and antimicrobial peptides (10–13). Human IL-17 family members display functional diversification. IL-17A and IL17-F induce IL-6, IL-8 and CXC chemokines (8, 14), while other human IL-17 forms are characterized by the induction of type-2 immunity (8, 15, 16).

Human IL-17 family members share low amino acid identity (35% average identity between human forms). Other mammals present the same IL-17 repertoire as humans, but because of the low sequence identities in these cytokines, it is rare to find sequence homologs among phyla. The identification of IL-17 sequences is based instead on the detection of their functional domain, which is characterized by unique structural features among the cysteine knot fold motifs and which implies different receptor interactions than other cytokines (8). This 4-cysteine conserved motif forms intrachain disulphide bonds and facilitates dimerization (17).

Other vertebrates, such as teleost fishes, present similar IL-17 families including 4-7 members (18). *Danio rerio* presents a family with homologous proteins for the A, F, C, and D forms but lacks the B and E forms (19). *D. rerio* instead possesses a novel ligand (IL-17N) characteristic of teleost fishes (20). This ligand was first identified in the fish *Takifugu rubripes* and encodes 7 isoforms (21). Equivalent repertoires have been found in other fishes (22–24), and functional studies have demonstrated their proinflammatory implications (25–27).

Invertebrate phyla possess IL-17 genes as well, although the knot fold motif is characterized by 2 extra cysteines located in positions characterized by two serine residues in chordates (28). Close to vertebrate species is the invertebrate chordate *Ciona intestinalis*, with three IL-17 genes activated by LPS inoculation (29). Several IL-17 and IL-17R genes have been subsequently identified in other invertebrates. Some show particularly large repertoires, as in the purple sea urchin *Strongylocentrotus purpuratus*, with 35 IL-17 genes and 2 receptors (30). IL-17 genes from the sea urchin were classified into 10 different subfamilies, showing functional diversification of their immune implications in the gut epithelium or in immune cells (31). In the mollusc *Octopus bimaculoides*, an expansion of IL-17 genes was also reported when 31 genes were identified in its genome (32).

Il-17 genes have also been identified in bivalves such as *Pinctada fucata martenssi* (33), *Crassostrea gigas* (34, 35), and mussels (*Mytilus galloprovincialis*), with 6 IL-17s and 3 IL-17Rs (35). IL-17 pathway components such as CIKS and TRAF6 have been traced in bivalves, and IL-17 bivalve genes are responsive to LPS or bacterial infections, demonstrating inflammatory functions through NF-kB signaling pathways (34–37).

Bivalves, specifically, *M. galloprovincialis*, represent an interesting species in terms of comparative immunology and genomics. These animals are intertidal filter feeders capable of dealing with the wide range of different pathogens that they are persistently exposed to, achieving extraordinary survival success (38–40). This resilience to pathogens and their invasive behavior make mussels an interesting model to investigate genomic adaptations that could explain these phenomena. The recently published *M. galloprovincialis* pangenome was characterized by a core set of 45,000 genes and a set of dispensable genes (20,000) which are affected by presence/absence phenomena and therefore could be absent in individual genomes. This phenomena, in addition to the great variability of specific gene families, could endow the species with a more diverse and enriched response capacity (41).

In bivalves, only a few cytokines have been identified in addition to IL-17, including TNF homologs, allograft inflammatory factor 1, macrophage migration inhibitory factor, and astakine, and the presence and modulation of an interferon-related pathway after viral infection could indicate the presence of an interferon-homologue cytokine not yet discovered (42–45). Additionally, some molecules initially known as antimicrobial peptides, such as myticin C in *M. galloprovincialis*, were revealed to have chemotactic functions as well as modulatory activity over some immune genes, acting as cytokines (46). Myticin C genes are subjected to massive variation and presence/absence phenomena in their variants in the mussel pangenome (47).

IL-17 inflammatory triggering functions seemed to be of importance in the mussel immune response in gills after the recognition of a bath bacterial infection (48). IL-17 genes were modulated in gills against the incoming infection, while there was no modulation in the defensive cells (the hemocytes), against a systemic infection with the same bacteria (48, 49). This might be another example of the role of IL-17 in the immune response of epithelial cells in different species (4, 31).

In the present work, we carried out a comparative genomics analysis of the IL-17 gene family across different phyla of interest. We were able to reveal potential ancestor species and conserved patterns in the evolution of IL-17 repertoires in certain animal classes. The genomic resources originating from the sequencing of 16 individuals to construct the mussel pangenome (41) allowed us to confirm that Il-17 did not show the presence/absence phenomena characteristic of numerous immune gene families previously described. Instead, there was a similar repertoire, in terms of expansion and variability, to those found in other marine invertebrates. Functional experiments and transcriptomic data for studying the implications of these cytokines in the mussel immune response against a waterborne infection in gills suggested functional specialization of the IL-17 family in mucosal immunity. The large IL-17 repertoires of certain marine invertebrates may be associated with different functions, but epithelial immune defenses appear to be conserved throughout evolution to humans.

## Material and Methods

### Identification and Analysis of IL-17 and IL-17R Sequences in the Mussel Genome

The *M. galloprovincialis* reference genome LOLA (41) was analyzed to identify the IL17-related sequences. Moreover, 15 resequenced mussel genomes (41) were also analyzed to identify putative IL-17 genes not included in the reference. These 16 genomes referred to five Galician female mussels (GALF1, GALF2, GALF3, and PURA apart from the mussel reference genome LOLA), five Galician males (GALM1, GALM2, GALM3, GALM6 and GALM11), three Italian females (ITAF1, ITAF2 and ITAF3) and three Italian males (ITAM1, ITAM2 and ITAM3). Analysis was mainly performed inside the CLC Genomics Workbench v.20 (Qiagen, Hilden, Germany).

First, the previously identified 6 IL-17 and 3 IL17-R mussel sequences (35) were used as queries and blasted (tblastn) with an e-value threshold of 1x10^-3^ against a blast database generated from the mussel reference genome. The resulting contigs were manually checked for the presence of complete open reading frames (ORFs), and the resulting genes were recovered. Then, a Pfam domain scan (hmmsearch algorithm) was performed on the predicted proteins from coding sequences of the mussel reference genome. Sequences containing IL17 domains (PF06083) or SEFIR domains (PF08357) were retrieved and compared with those obtained from the BLAST approach to remove redundancies. A final list of IL17 and IL17-R sequences obtained from the mussel reference genome with both methods was generated.

This list was blasted (blastn) against the 15 resequenced mussel genomes (41). All genomic contigs corresponding to IL17 and IL17-R sequences were retrieved from each genome. Blast hits were analyzed as described above, and the obtained ORFs were also translated to their encoded proteins. The final list of IL-17 and IL-17R genes obtained from the 16 mussel genomes was submitted to CD-HIT (50–52), and only unique sequences were retained. The unique nucleotide sequences cleared in the exon coding for their protein domain (IL17 or SEFIR) were aligned and submitted to model testing using CLC Genomics Workbench to test the best-fitting molecular model of evolution for these sequence sets. Jukes–Cantor (53) was obtained as the best-fitting model for IL-17 sequences, while GTR+G with a gamma-distributed rate of variation across sites (54) was the model for the receptors. IL-17 and IL-17R unique sequences were submitted to independent Markov chain Monte Carlo analyses run in Mr. Bayes v3.2.7a (55). The substitution model obtained for each case was considered, and analyses were run with a sampling frequency of 1,000 and a burn-in of 25% sampled trees until the average standard deviations of the split frequencies were ≤ 0.05. The analyses were run for 4 million generations with IL-17 and 350,000 generations with the receptors. The obtained consensus trees were graphically rendered using FigTree (56) and iTOL (57).

Clustering of mussel IL17 and IL17-R sequences was performed based on phylogenetic analyses. CD-HIT analyses with a sequence identity cut-off of 0.8 (80% homology) resulted in the same clusters. Primary sequence analysis was performed with a representative sequence of each cluster or isoform. Conserved domains were analyzed with HMMER 3 (58), transmembrane domains were analyzed using the TMHMM Server v. 2.0 (59, 60) and signal peptides were analyzed using the SignalP-5.0 Server (61, 62).

The genomic location in the reference genome scaffolds, the gene structure and the neighboring genes were also analyzed for the IL-17 and IL-17R genes present in the reference genome.

### Phylogenetic Distribution of IL-17 Repertoires Among Species

Genome assemblies and their encoded protein databases were downloaded from the NCBI for several species of interest across different phyla (IDs are displayed). The analysis strategy was equivalent to that performed in mussels.

For each species, previous IL-17 sequences were downloaded from the Pfam database and used as a query seed list that was blasted in a tblastn search against the corresponding genome assembly (e-value threshold of 1x10^-3^). For species with no previous sequences with IL17 domains, sequences of the phylogenetically closest species were downloaded from Pfam and used as queries. BLAST hits were analyzed, and the potential ORFs and protein sequences were retrieved. In a complementary way, a Pfam domain scan (hmmersearch) was conducted for the encoded proteins of each downloaded genome. CD-HIT was used to remove redundant sequences obtained from both methods for each species (identity cut-off of 1).

The exceptions to this pipeline were *Homo sapiens* and *Mus musculus*, since their IL17 gene repertoires were retrieved directly from the UniProt database (Q16552, Q9UHF5, Q9P0M4, Q8TAD2, Q9H293, Q96PD4, Q62386, Q9QXT6, Q8K4C5, A0A0B4J1G4, Q8VHH8, and Q7TNI7).

The IL-17 families from each species were clustered with the same 80% homology criteria. The evolutionary cladogram of the studied species was built using the TimeTree resource of MEGA-X Software (63).

A subset of selected species was submitted to MEME Suite 5.1.1 (64) to identify conserved motifs among their IL17 sequences.

Several Mytilidae species were also analyzed by searching for repertoires homologous to those discovered in *Mytilus galloprovincialis.* Genomic or transcriptomic assemblies were downloaded from the NCBI-SRA database, depending on the material available (IDs are displayed). A list containing representative sequences from each IL-17 isoform found in *M. galloprovincialis* was used as a query in a BLASTn search against these Mytilidae assemblies (e-value threshold of 1x10^-3^). BLAST hits were analyzed, and the potential ORFs were retrieved and translated to the protein sequences, confirming the presence of the IL-17 domain. The nucleotide coding sequences were submitted to phylogenetic neighbor joining analysis using the Jukes–Cantor substitution model (the best fitting molecular model of evolution for these sequences).

### Expression of IL-17 and IL17-R in Transcriptomic Studies

Transcriptomic data were recovered from two different experiments performed with individual *Mytilus galloprovincialis* mussels, including a gill transcriptome obtained after 24 h of waterborne exposure to an infection with *Vibrio splendidus* (48) and a transcriptome obtained from hemocytes 24 h after injection with the same bacteria, with the goal of establishing a systemic infection (49). Assemblies were constructed as explained in Saco et al. (48) and in Rey-Campos et al. (49), and reads from each transcriptome are accessible through PRJNA638821 and PRJNA466718, respectively. IL-17 and IL-17R protein sequences from the isoforms of the reference genome were used in a tblastn search against those assemblies. Contigs corresponding to the blast hits were obtained and aligned to verify their classification within a specific IL17/IL17-R cluster or isoform. Transcriptomic expression was retrieved in transcripts per million (TPM).

### Mussel Bath Infection and Tissue Sampling

Adult *Mytilus galloprovincialis* mussels were obtained from a commercial shellfish farm (Vigo, Galicia, Spain) and acclimatized in tanks with open-circuit filtered seawater (FSW) at 15°C with aeration. Mussels were submitted to a waterborne exposure to 10^8^ CFU/ml *Vibrio splendidus* (reference strain LGP32), while control mussels were maintained in FSW.

Samples from hemocytes and gills were obtained after 24h of waterborne exposure to the bacterial infection. Hemolymph was withdrawn from the posterior adductor muscle using a 0.5 mm-diameter (25 G) disposable needle through a hole made in the shell. Hemolymph was centrifuged at 4°C at 1000xg for 20 min, and the hemocyte pellet was suspended and homogenized in the same volume of FSW. Gills were sampled and homogenized in FSW, as well.

### RNA Extraction, cDNA Synthesis and Quantitative Real-Time PCR (qRT-PCR)

RNA was extracted from individual triplicates of infected and control hemocyte and gill samples. RNA extraction was carried out using the Maxwell 16 LEV simply RNA kit (Promega, Madison, Wisconsin, United States), and its concentration and purity were measured with a NanoDrop ND1000 spectrophotometer (NanoDrop Technologies, Wilmington, Delaware, United States). cDNA was synthetized by reverse transcription from 300 ng of total RNA from each sample using an NZY First-Strand cDNA Synthesis Kit (Nzytech, Lisboa, Portugal).

The gene expression of several mussel IL17 and IL17-R genes (**Supplementary Table ST1**) was analyzed in a Step One Plus qPCR System (Applied Biosystems, Foster City, California, United States). The selected isoforms were those showing expression in the previously mentioned gill transcriptome. The cycling conditions were 95°C for 10 min, followed by 40 cycles of 95°C for 15 s and 60°C for 30 s. Reactions were performed in technical triplicates, and the relative expression was normalized using highly stable 18S gene expression as a housekeeping gene, following the delta-delta-CT method (65). Significant differences were analyzed (t-test, p-value ≤ 0.05).

### *In Situ* Hybridization in Tissue Samples

*In situ* hybridization (ISH) was carried out to localize the expression of the IL17-3 gene in gill histology preparations with digoxigenin-labelled IL17-3-specific RNA probes. This isoform was selected because it was one of the most expressed isoforms (35), and it was modulated in the analyzed gill transcriptome. To obtain the probes, mussel cDNA was amplified in two different PCRs combining pairs of IL17-3 primers, one of which was a normal primer at 10 mM, and the other included the attached SP6/T7-corresponding probe sequence (**Supplementary Table ST1**) at 100 mM (Fsp6 + R; F + RT7). Cycling conditions were set to 94°C 5 min, 35 cycles of 94°C 30 s + 60°C 30 s + 72°C 1 min and a final step of 72°C 7 min. PCR products were filtered and concentrated using Amicon Ultra 0.5-10k devices following kit instructions (Sigma-Aldrich, St. Louis, Missouri, United States). Digoxigenin-labelled IL17-3-specific RNA probes, both antisense (As) and sense (S; control), were obtained by *in vitro* transcription of 1 µg of purified cDNA from each PCR product using the DIG RNA labelling kit (SP6/T7) and following the kit instructions (Roche, Basel, Switzerland). Finally, both sense and antisense probes were purified using SigmaSpin Sequencing Reaction Clean-Up (Sigma-Aldrich). After each step of the probe preparation process, the cDNA/RNA concentration was measured using a NanoDrop ND1000 spectrophotometer, and electrophoresis gels were run to check the specificity.

ISH assays were performed simultaneously on two sets (As and S) of duplicate slides, each with serial sections of gill from the same animal. Briefly, mussels were fixed using formaldehyde and embedded in paraffin. Seven-micrometer cut sections were placed on polylysine-coated glass slides (Thermo Scientific, Waltham, Massachusetts, United States), dried and submitted to deparaffinization and rehydration (passes with xylene, 100%, 95% and 70% ethanol and DEPC-treated ddH_2_O). After incubation with DEPC-PBS (2x5 min) and DEPC-PBS with 100 mM glycine (2x5 min), tissue sections were treated with DEPC-PBS 0.3% Tween-20 (15 min) and washed again with DEPC-PBS (2x5 min). Afterward, a permeabilization step with 20 µg/ml proteinase K in TE buffer (100 mM Tris-HCl, 50 mM EDTA, pH 8) at 37°C°C for 30 min was performed. Finally, sections were postfixed with PBS-PFA 4% (5 min, 4°C), washed and acetylated for 20 s with 20% acetic acid.

Prehybridization was carried out by incubating tissue sections for 90 min at 37°C in a humid chamber with hybridization buffer (40% formamide, 10% dextran sulphate, 1x Denhardt’s solution, 4x SSC, 10 mM DTT, 1 mg/ml yeast t-RNA, 1 mg/ml salmon sperm DNA). Hybridization buffer was added again and supplemented with 50 ng of the corresponding digoxigenin (DIG)-labelled RNA probe for each slide. Sections were incubated at 42°C overnight in a humid chamber.

After incubation, the samples were washed at 37°C in 2x SSC (2x15 min) and 1x SSC (2x15 min). To digest any single-stranded nonspecifically bound probes, incubation for 30 min at 37°C with 20 µg/ml RNaseA in NTE buffer (500 mM NaCl, 10 mM Tris, 1 mM EDTA, pH 8) was performed. The slides were again washed at 37°C in 0.1x SSC (2x30 min), incubated in a buffer containing 100 mM Tris-HCl and 150 mM Nacl at pH 7.5 (2x10 min), then incubated for 30 min in a blocking solution with 0.1% Tween-20 and 2% normal sheep serum added to the buffer, and finally incubated for 2 h in a humid chamber after supplementing the buffer with 0.1% Tween-20, 1% normal sheep serum and a dilution 1:250 of anti-DIG-alkaline phosphatase antibody [Fab fragments]. After two washes with the buffer, the slides were incubated for 10 min with 100 mM Tris-HCl, 100 mM NaCl, and 50 mM MgCl_2_ at pH 9.5, and then 1 mM levamisole, 4.5 µl/ml nitroblue tetrazolium (NBT) and 3.5 µl/ml 5-bromo-4-chloro-3-indolyl-phosphate (BCIP) solutions were added. Finally, the slides were covered with this solution and incubated in darkness in a humid chamber until an easily visible color was found due to alkaline phosphatase detection. The color reaction was stopped with 10 mM Tris-HCl and 1 mM EDTA at pH 8.1, and the slides were washed and finally mounted using DPX.

## Results

### Screening and Classification of the Mussel IL-17 and IL-17R Repertoire

**Figure 1:**
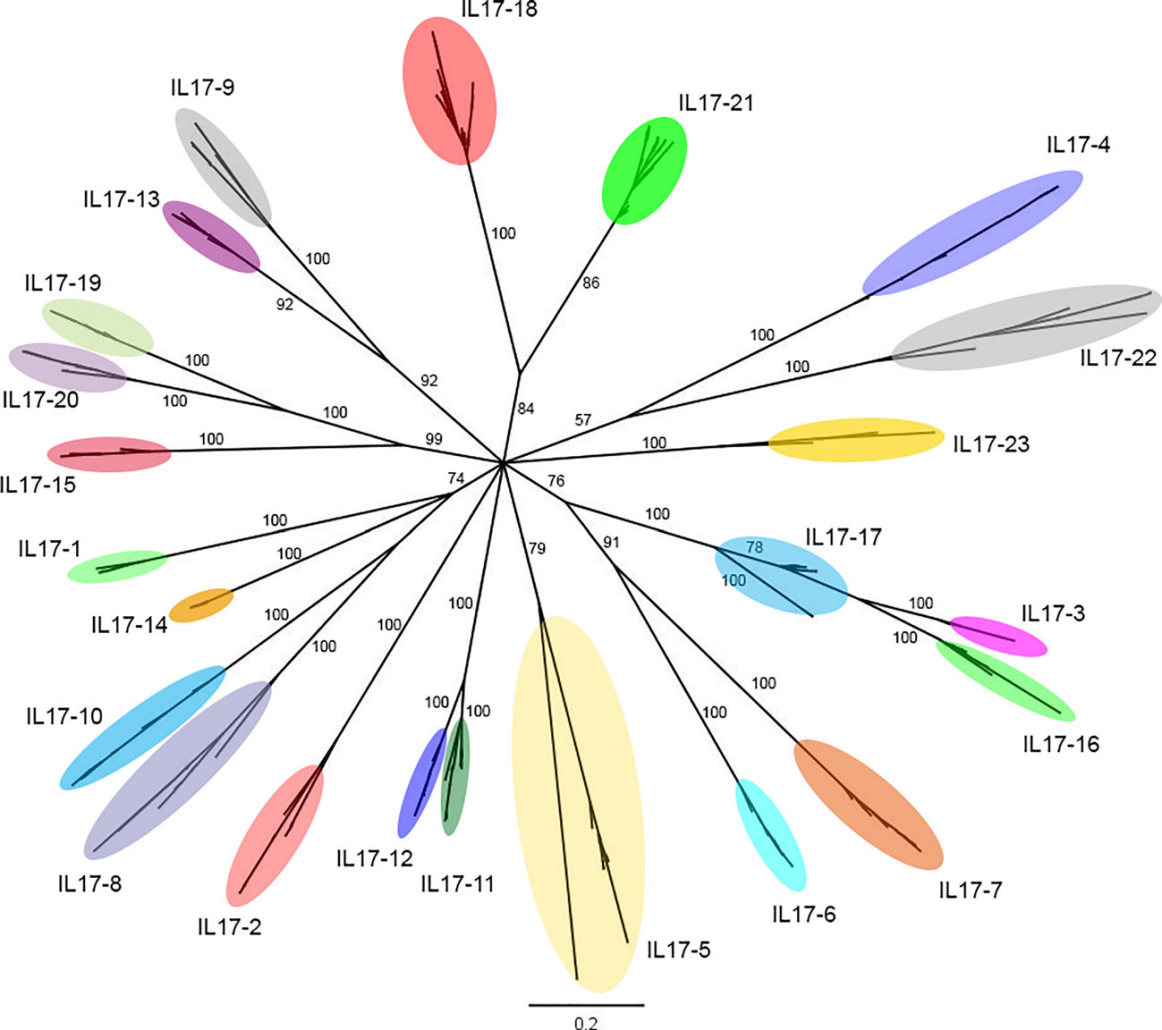
Phylogenetic analysis of the 379 unique IL-17 sequences obtained from the 16 mussel genomes. The 379 unique IL-17 sequences retrieved from 16 mussel genomes were submitted to phylogenetic analysis with Bayesian phylogenetic inference using the Jukes and Cantor (JC) evolution model. The 23 different IL-17 clusters or isoforms obtained are indicated with ellipses that englobe all their unique variants. The figure shows branch posterior probabilities. It can be seen how isoforms IL17-3 and IL17-16 would be diverging from IL17-17, since the branch of the latter contains both isoforms. The distinction between these isoforms was maintained due to the homology criterion and to expression evidence.

In total, 552 sequences of IL-17 were identified after applying the screening pipeline to 16 mussel genomes (41), resulting in 379 unique sequences that are deposited in **Supplementary Data 1**. These sequences were clustered based on an identity percentage threshold of 80%, yielding 23 clusters. The Bayesian tree of the 379 unique sequences displayed diversification in the same 23 clusters (**Figure 1**). Six out of 23 IL-17 forms (1-6) were already described by Rosani et al. (35). The new forms were named using the numbers 7 to 23.

As shown in **Table 1**, in each genome, the number of IL-17 genes varied between 29 and 42, with an average repertoire of 34 genes per mussel. These variants were classifiable into the 23 defined clusters. There was no presence/absence phenomenon at the isoform level. Instead, every individual mussel had its own unique sequences belonging to the 23 clusters, characterized by variations in specific positions and resulting in a large number of 379 nonshared sequences. Isoforms IL17-18 and IL17-21 were the most diverse, presenting up to 5 different variants in some mussels.

**Table 1:** Sequences present in each of the 16 analyzed mussel genomes that correspond to the different IL-17 and IL-17R isoforms.

	Sequenced mussel genomes															
IL17	LOLA	GALF1	GALF2	GALF3	GALM1	GALM2	GALM3	GALM6	GALM11	ITAF1	ITAF2	ITAF3	ITAM1	ITAM2	ITAM3	PURA
IL17-1	1	1	1	1	3	1	2	1	2	1	1	1	2	1	2	1
IL17-2	2	1	2	2	1	1	1	2	1	2	2	1	1	1	1	1
IL17-3	2	1	1	1	1	1	1	1	1	1	1	1	1	2	2	1
IL17-4	2	1	1	1	1	1	1	2	2	2	1	1	2	2	2	1
IL17-5	1	1	1	1	1	1	1	2	2	1	1	1	2	2	1	1
IL17-6	2	2	2	1	1	2	2	2	2	1	1	1	1	1	1	2
IL17-7	2	1	2	1	2	2	2	1	1	2	1	2	2	1	1	1
IL17-8	1	1	2	1	1	2	2	2	2	1	1	2	2	2	2	1
IL17-9	1	1	1	1	2	1	1	1	1	1	1	1	1	1	1	1
IL17-10	1	1	1	1	1	1	1	2	1	1	1	1	1	1	1	1
IL17-11	2	3	2	2	4	2	3	2	2	2	2	1	2	2	2	2
IL17-12	1	2	2	1	1	1	1	2	1	–	–	1	1	2	1	1
IL17-13	1	2	1	2	1	1	1	1	1	1	1	1	1	1	1	1
IL17-14	1	1	1	1	2	1	1	2	1	2	1	2	2	2	2	1
IL17-15	1	1	1	2	1	1	2	1	1	1	1	1	1	1	1	1
IL17-16	1	1	2	1	2	1	1	2	1	–	1	1	1	1	1	1
IL17-17	1	2	1	2	2	1	1	1	1	2	2	2	1	1	2	1
IL17-18	3	4	4	4	5	4	3	4	4	3	3	3	4	2	2	2
IL17-19	1	1	2	1	1	1	1	2	1	1	1	1	1	1	1	1
IL17-20	1	1	2	2	2	2	2	2	1	2	2	1	1	1	2	2
IL17-21	3	5	5	1	3	2	5	5	5	2	4	2	2	5	3	3
IL17-22	1	1	1	1	1	1	1	1	1	1	1	1	1	1	1	1
IL17-23	1	1	1	1	1	1	2	1	1	1	1	1	1	1	1	1
**Total**	33	36	39	32	40	32	38	42	36	31	31	30	34	35	34	29
**IL17R**	**LOLA**	**GALF1**	**GALF2**	**GALF3**	**GALM1**	**GALM2**	**GALM3**	**GALM6**	**GALM11**	**ITAF1**	**ITAF2**	**ITAF3**	**ITAM1**	**ITAM2**	**ITAM3**	**PURA**
IL17R-A	2	1	2	2	2	1	1	1	1	1	1	1	2	1	1	1
IL17R-B	1	1	1	1	1	–	1	1	1	1	1	1	1	1	1	1
IL17R-C	1	1	1	1	1	2	2	1	1	2	1	1	1	1	1	1
IL17R-D	2	1	2	2	1	1	2	1	1	2	1	2	1	1	1	1
IL17R-E	1	2	2	2	1	1	1	2	1	1	1	1	1	1	1	1
IL17R-F	1	1	2	1	1	–	2	1	1	1	2	1	1	1	1	1
**Total**	8	7	10	9	7	5	9	7	6	8	7	7	7	6	6	6

Considering this conservation at the isoform level, representative sequences were selected from the reference genome for each isoform (listed in **Supplementary Data 2**). As indicated in **Table 2**, they ranged from 126 to 221 amino acids, always with an IL-17-defined domain towards the C-terminus. The signal peptide was predicted in 12 out of 23 consensus isoforms. The multiple sequence alignment of the conserved domains is displayed in **Supplementary Figure S1**. Every isoform presented 6-8 cysteine residues in the functional domain, which would conform to the at least three disulphide bonds typical of invertebrate IL-17s. Isoforms 18 and 21 showed only 4 conserved cysteine residues, and therefore, they could form only the two disulphide bonds characteristic of chordate species.

**Table 2:** Sequence and domain information of the 23 isoforms of mussel IL-17.

IL17 cluster representatives	Predicted Protein Length	Signal peptide Sec/SPI Likelihood	Predicted signal peptide cleavage site	IL17 domain e-value (HMMER3)	IL17 domain position
**IL17-1**	194	0.6047	27 to 28	1.1e-13	102 to 185
**IL17-2**	192	0.9448	18 to 19	1.2e-16	89 to 168
**IL17-3**	194	0.9842	23 to 24	3.7e-12	94 to 169
**IL17-4**	165	0.9053	26 to 27	3.4e-17	82 to 157
**IL17-5**	221	0.5281	23 to 24	2.2e-12	95 to 173
**IL17-6**	192	0.7977	24 to 25	8.8e-14	94 to 170
**IL17-7**	192	0.937	22 to 23	4.4e-13	95 to 170
**IL17-8**	134	0.0033	-	2.4e-13	46 to 119
**IL17-9**	169	0.0008	-	2.4e-19	60 to 134
**IL17-10**	180	0.9751	20 to 21	1.7e-14	85 to 162
**IL17-11**	179	0.0021	-	4.1e-17	91 to 166
**IL17-12**	194	0.939	22 to 23	8.1e-19	106 to 181
**IL17-13**	198	0.4734	-	1.6e-19	87 to 161
**IL17-14**	185	0.9211	17 to 18	1.7e-16	82 to 168
**IL17-15**	185	0.0654	-	6.5e-14	85 to 167
**IL17-16**	194	0.4703	-	1.4e-11	94 to 169
**IL17-17**	190	0.9332	19 to 20	4.4e-12	90 to 166
**IL17-18**	126	0.0043	-	3.3e-08	43 to 116
**IL17-19**	133	0.0019	-	2.8e-15	53 to 127
**IL17-20**	137	0.001	-	5e-14	55 to 131
**IL17-21**	116	0.0033	-	6.4e-12	41 to 114
**IL17-22**	174	0.0009	-	1.9e-17	85 to 169
**IL17-23**	133	0.996	20 to 21	3.6e-05	53 to 131

**Figure 2:**
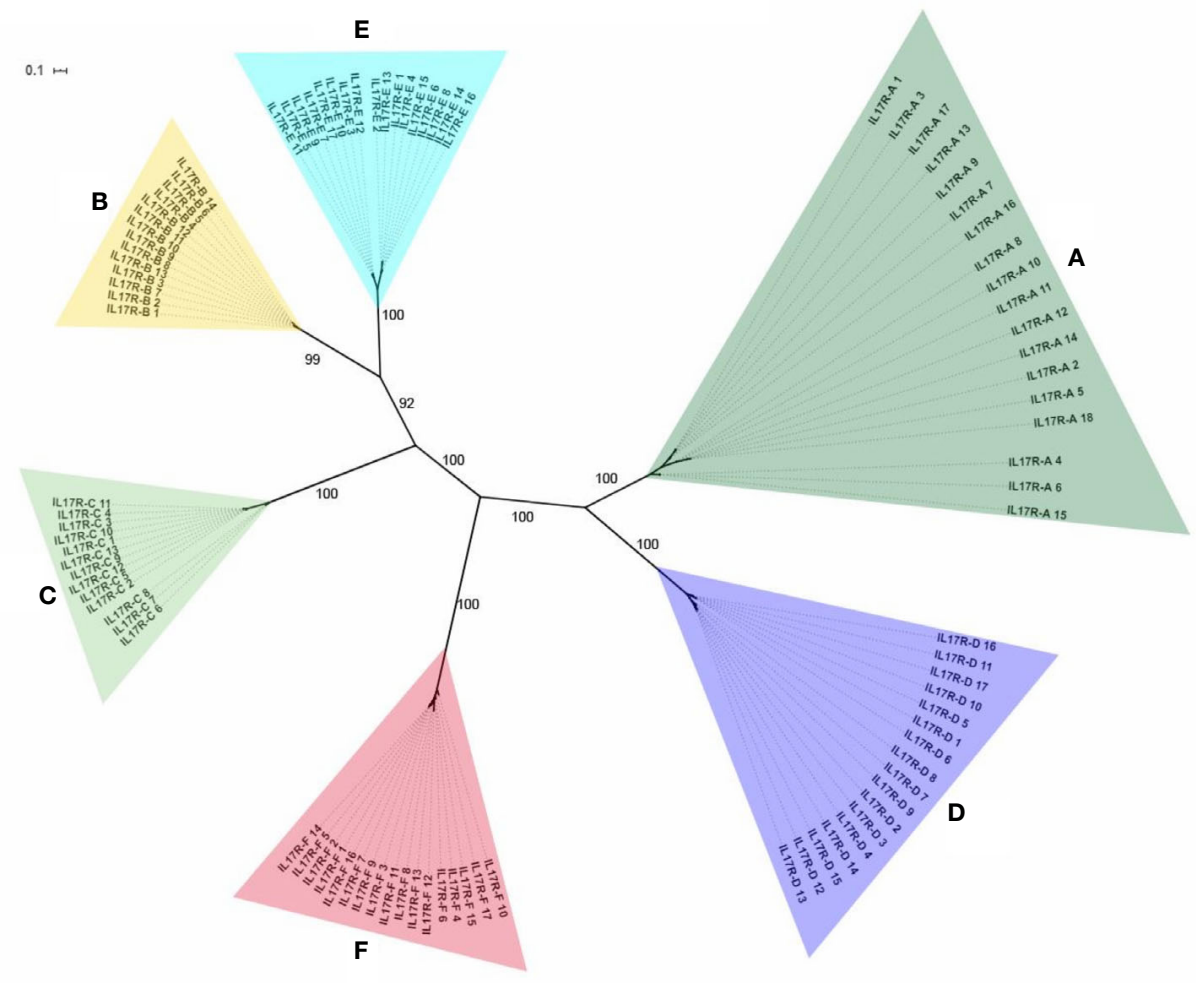
Phylogenetic analysis of the 96 unique IL-17R sequences obtained from the 16 mussel genomes. The 96 unique IL-17R sequences retrieved from 16 mussel genomes were submitted to phylogenetic analysis with Bayesian phylogenetic inference using the GTR+G evolution model. The 6 different IL-17 clusters or isoforms obtained are indicated. The figure shows branch posterior probabilities.

In total, 115 nucleotide IL-17 receptor sequences were retrieved from the 16 mussel genomes, resulting in 96 unique sequences (deposited in **Supplementary Data 3**). These unique sequences were clustered into 6 isoforms (identity percentage threshold of 80%) and submitted to phylogenetic analysis, which resulted in the same 6 isoforms or clusters (**Figure 2**). Again, as with the IL-17 genes, variability was enormous inside each cluster, but no presence/absence phenomenon was observed at the isoform level (**Table 1**). Three out of six clusters were already described by Rosani et al. (35), which were A, B and C. The three new clusters were named D, E and F to maintain the nomenclature system already in use for mussel, but this is not related to possible vertebrate orthologous. Representative sequences for each isoform were retrieved from the reference genome (**Supplementary Data 4**). SEFIR domains were conserved, as were the transmembrane regions that separate extracellular and cytoplasmic domains, indicating that these receptors would be functional in terms of signaling (**Table 3**).

**Table 3:** Sequence and domain information of the 6 isoforms of mussel IL-17R.

IL17 receptor isoforms in LOLA genome	Predicted Protein Length	Transmembrane region (TMHMM 2.0)	SEFIR domain e-value (HMMER3)	SEFIR domain position
**IL17R-A**	636	221 to 243	7.8e-12	286 to 437
**IL17R-B**	727	350 to 372	3.4e-16	401 to 542
**IL17R-C**	566	13 to 35	9.9e-30	71 to 223
**IL17R-D**	778	353 to 375	6.2e-13	429 to 580
**IL17R-E**	685	353 to 375	9.7e-12	407 to 549
**IL17R-F**	874	386 to 409	6.6e-12	467 to 602

### IL-17 Gene Families Throughout Evolution

**Figure 3:**
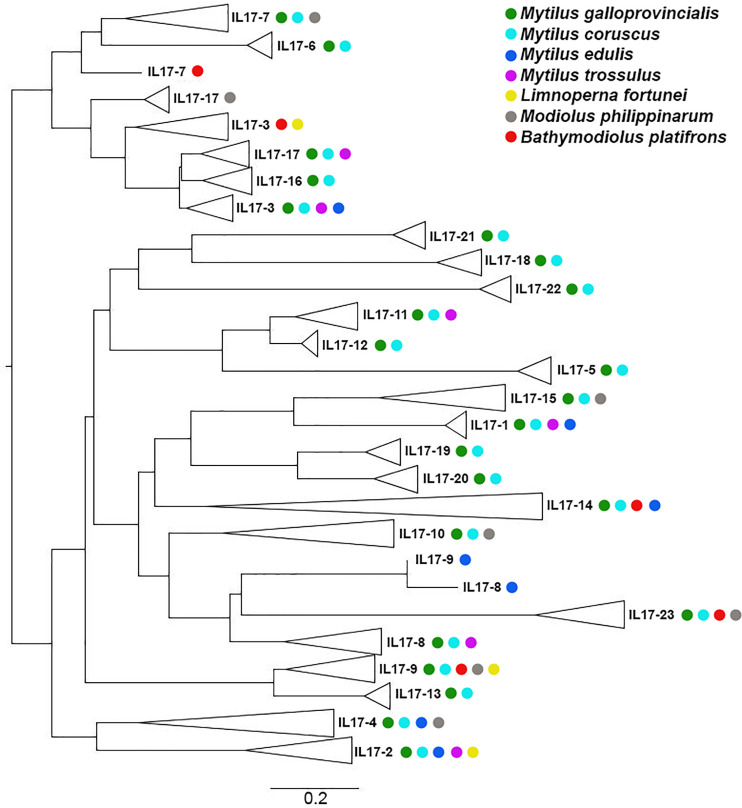
Phylogenetic analysis of the IL-17 families found in Mytilidae species. Homologous sequences to the *M. galloprovincialis* IL-17 isoforms were found in several Mytilidae species and submitted to neighbor joining analysis using the Jukes–Cantor substitution model. Clustering was based on isoform homologs, and species are indicated with colors.

Genomic or transcriptomic assemblies from different Mytilidae species were scanned for IL-17 sequences, and those matching the requirements were retrieved (**Table 4**). Every IL-17 gene from the studied Mytilidae species was homologous to a certain isoform of *M. galloprovincialis*. Hence, the resulting phylogenetic tree was clustered by isoform and not by species (**Figure 3**). *Mytilus coruscus* was the closest species, presenting every IL-17 isoform found in *M. galloprovincialis*. The most variable isoforms, IL17-18 and IL17-21, were characterized by two different genes each in the *M. coruscus* genome. Most isoforms were only conserved with homologous sequences in *M. galloprovincialis* and *M. coruscus*, while others, such as IL17-2, IL17-3, IL17-9 or IL17-14, were quite conserved across the different Mytilidae species studied.

**Table 4:** Homologous sequences to the *Mytilus galloprovincialis* IL-17 isoforms found in other analyzed Mytilidae species.

Species	Assembly	Project code	Contigs count	Technology	IL17 Forms
*Mytilus coruscus*	Genomic	PRJEB33342	10484	PromethION DNA sequencer	25
*Mytilus californianus*	Transcriptomic	PRJNA375125	59027	Illumina NextSeq 500	0
*Mytilus edulis*	Genomic	PRJNA525607	353272	Illumina HiSeq	7
*Perna viridis*	Transcriptomic	PRJNA478494	73264	Illumina HiSeq	0
*Limnoperna fortunei*	Genomic	PRJNA330677	61104	Illumina NextSeq 500; PacBio	3
*Mytilus trossulus*	Transcriptomic	PRJNA525608	437716	Illumina HiSeq	6
*Modiolus philippinarum*	Genomic	PRJNA328544	74573	Illumina HiSeq	9
*Bathymodiolus platifrons*	Genomic	PRJNA328542	272497	Illumina HiSeq	5

**Figure 4:**
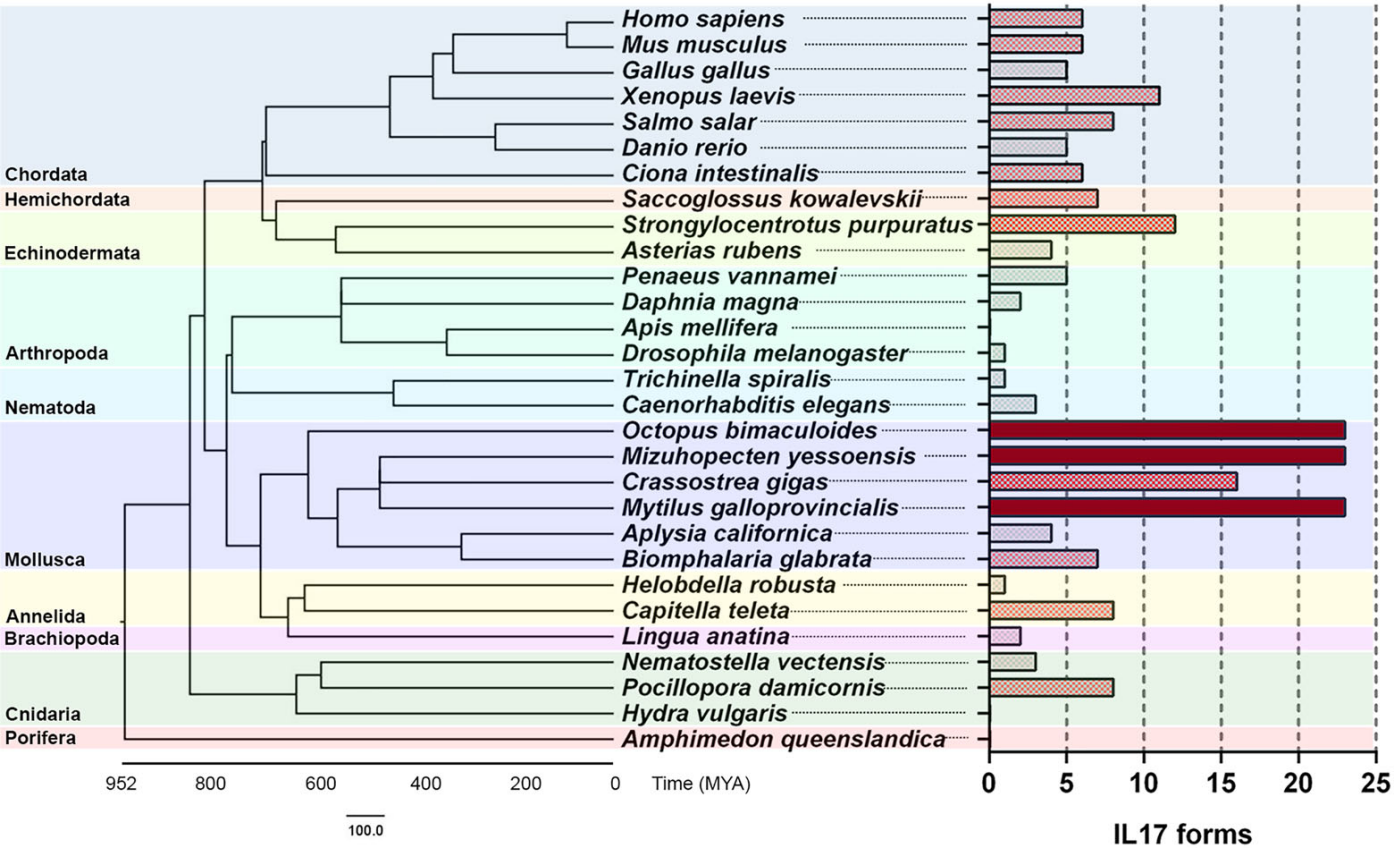
Comparative genomics analysis of IL-17 gene families throughout evolution. IL-17 repertoires were analyzed in several species from different phyla and clustered with an 80% homology criterion. The evolutionary cladogram is presented, along with the extension of the IL-17 family from each species.

Species belonging to different animal phyla were also analyzed in search of their IL-17 gene repertoires (**Table 5**). The results revealed putative ancestor species and expanded IL-17 repertoires in certain invertebrate species (**Figure 4**). We could not identify any putative IL-17 gene in the sponge *Amphimedon queenslandica* or in the first divergent cnidarian analyzed, *Hydra vulgaris*. Nevertheless, the two other cnidarians analyzed displayed relatively high numbers of IL-17 sequences, reaching 11 genes in *Pocillopora damicornis*, which were classified into 8 clusters. In the present work, Cnidaria emerged as the most ancient divergent phylum with IL-17 gene families. Brachiopoda and Annelida species included in the analysis did show different repertoires present in all species, as did the studied Nematoda species (*T. spiralis* and *C. elegans*). Concerning Mollusca, there was a common evolutionary pattern, with large expanded IL-17 repertoires compared to other phyla, as shown in **Figure 4**. This was observed in all the marine bivalves included in the study (*M.* yessoensis, *C. gigas* and *M. galloprovincialis*) and in the marine cephalopod *Octopus bimaculoides*, as reported by Albertin et al. (32). These species surpassed the 30 genes each and clustered into 23 isoforms, except *C. gigas*, with 24 genes and 16 clusters (**Table 5**). Analyzed mollusc species from the Gastropoda class (*Biomphalaria glabrata* and *Aplysia californica*) did not show these expanded IL-17 families.

**Table 5:** IL-17 genes and isoforms found in the analyzed species from different phyla.

Phylum	Species	NCBI Genome ID	RefSeq/GenBank assembly accession	IL-17 unique sequences	IL-17 clusters (80%)
Porifera	*Amphimedon queenslandica*	2698	GCF_000090795.1	–	–
Cnidaria	*Hydra vulgaris*	12836	GCF_000004095.1	–	–
Cnidaria	*Pocillopora damicornis*	22550	GCF_003704095.1	11	8
Cnidaria	*Nematostella vectensis*	230	GCF_000209225.1	3	3
Brachiopoda	*Lingua anatina*	38582	GCF_001039355.2	4	2
Annelida	*Capitella teleta*	15118	GCA_000328365.1	11	8
Annelida	*Helobdella robusta*	15112	GCF_000326865.1	1	1
Mollusca - Gastropoda	*Biomphalaria glabrata*	357	GCF_000457365.1	8	7
Mollusca - Gastropoda	*Aplysia californica*	443	GCF_000002075.1	4	4
Mollusca - Bivalvia	*Mytilus galloprovincialis*	12190	GCA_900618805.1	33	23
Mollusca - Bivalvia	*Crassostrea gigas*	10758	GCF_902806645.1	24	16
Mollusca - Bivalvia	*Mizuhopecten yessoensis*	12193	GCF_002113885.1	31	23
Mollusca - Cephalopoda	*Octopus bimaculoides*	41501	GCF_001194135.1	36	23
Nematoda	*Caenorhabditis elegans*	41	GCF_000002985.6	4	3
Nematoda	*Trichinella spiralis*	238	GCF_000181795.1	1	1
Arthropoda - Insecta	*Drosophila melanogaster*	47	GCF_000001215.4	1	1
Arthropoda - Insecta	*Apis mellifera*	48	GCF_003254395.2	–	–
Arthropoda - Crustacea	*Penaeus vannamei*	10710	GCF_003789085.1	6	5
Arthropoda - Crustacea	*Daphnia magna*	10953	GCF_003990815.1	2	2
Echinodermata	*Asterias rubens*	83686	GCF_902459465.1	4	4
Echinodermata	*Strongylocentrotus purpuratus*	86	GCF_000002235.5	26	12
Hemichordata	*Saccoglossus kowalevskii*	359	GCF_000003605.2	9	7
Chordata - Tunicata	*Ciona intestinalis*	49	GCF_000224145.3	7	6
Chordata - Actinopterygii	*Salmo salar*	369	GCF_000233375.1	10	8
Chordata - Actinopterygii	*Danio rerio*	50	GCF_000002035.6	5	5
Chordata - Amphibia	*Xenopus laevis*	81	GCF_017654675.1	18	11
Chordata - Aves	*Gallus gallus*	111	GCF_016699485.2	5	5
Chordata - Mammalia	*Mus musculus*	52	GCF_000001635.27	6	6
Chordata - Mammalia	*Homo sapiens*	51	GCF_000001405.39	6	6

Among Arthropoda species, crustaceans such as *Penaeus vannamei* and *Daphnia magna* contained IL-17 repertoires. IL-17 has not been previously described in Insecta, with no reports of these genes in the class (28). However, one sequence with the typical structure of an interleukin, with 218 amino acids, a signal peptide and with the C-terminal IL-17 domain (e-value of 4.7e-05) emerged from the *Drosophila melanogaster* genome in our study. This did not occur in the other analyzed insect, *Apis mellifera*.

The echinoderm *Strongylocentrotus purpuratus* displayed a large repertoire, which was closer to that of the studied marine molluscs. We were able to find 26 sequences in its genome. However, the presence of up to 35 IL-17 sequences in the genome of this species has been reported, and the repertoire must be larger than the revealed from the assembly with which we worked (30, 31). The whole repertoire of 35 genes is reported to cluster into 10 subfamilies or isoforms, which is more consistent with the isoforms that we found (31). The identified sequences may be suitable to represent the diversity of *S. purpuratus* in this study, since the comparative genomics analysis was focused on an approach based on isoforms instead of genes. As in Mollusca, not all echinoderms displayed expanded IL-17 repertoires, since only 4 genes were found in *Asterias rubens*.

We also described, for the first time, an IL-17 repertoire in hemichordates, in particular, *Saccoglossus kowalevskii*. IL-17 had not been previously reported in this phylum. Focusing on Chordata species, we studied the invertebrate *Ciona intestinalis* and some vertebrate species, finding families with similar numbers of IL-17 isoforms (± 6) and being the amphibian *Xenopus laevis* the one with the richest repertoire.

Due to the low sequence identities that IL-17 sequences display, homologous forms can be found only in phylogenetically close species, as seen with Mytilidae or typically reported among mammals or vertebrates. This can be observed in the phylogenetic analysis of IL-17 genes from different phyla, as shown in **Supplementary Figure S2**. Genes from different species do not share high sequence homology but maintain a conserved structure. The IL-17 domain emerges, supported by the mentioned cysteine pattern and conserved motifs, as recurrent CPW/SPW in invertebrates and chordates, respectively (**Supplementary Figure S3**).

**Figure 5:**
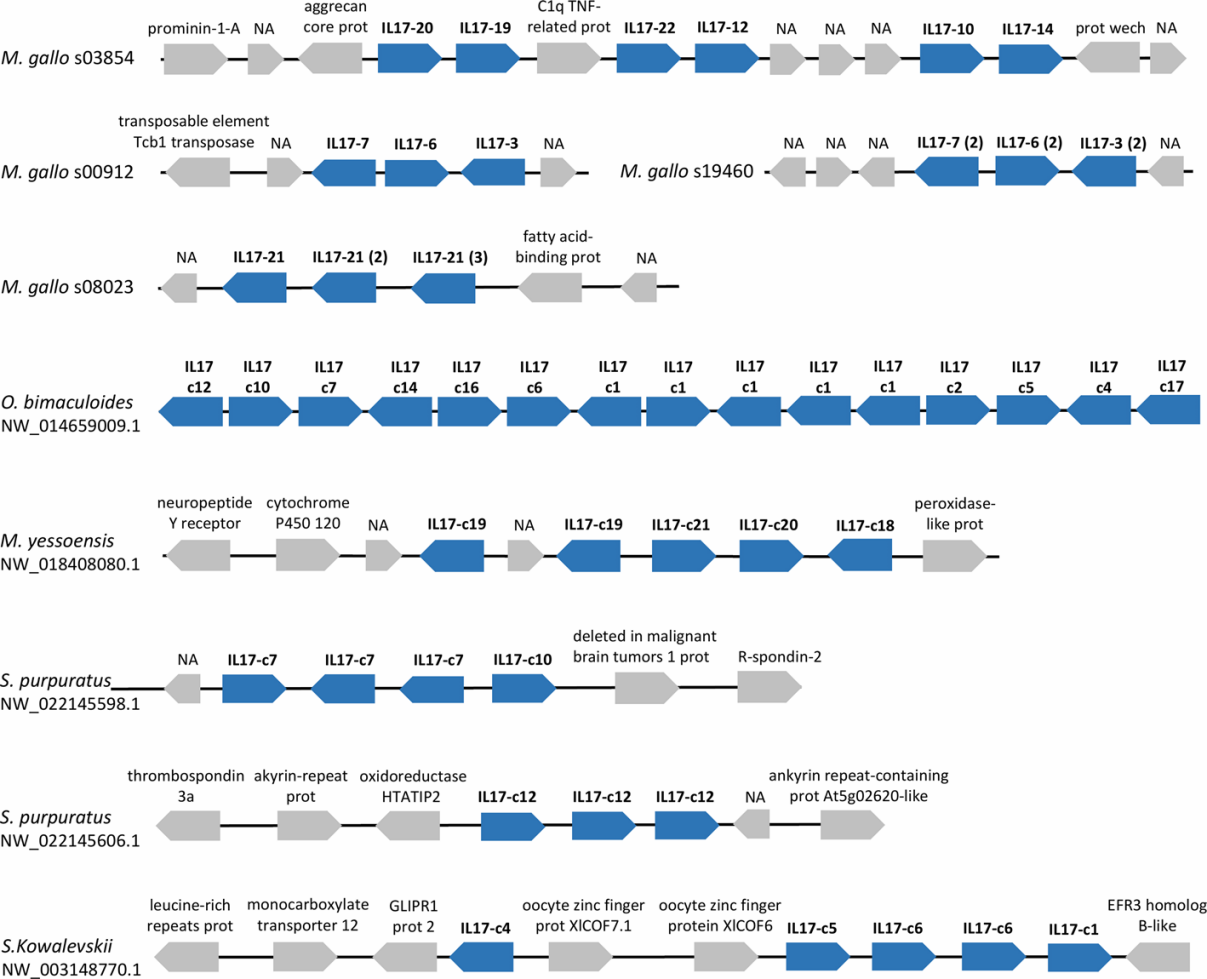
IL-17 genes often appear in tandem repetitions in the genomes of marine invertebrates with expanded IL-17 repertoires. Examples of several scaffolds with IL-17 tandem duplications are presented for these species. IL-17s are indicated with the name of the cluster to which they belong in our analysis. Tandem repetitions can occur with genes from the same or different isoforms. Neighbor genes are represented with their functional annotations.

The study of possible synteny conservation between mussel isoforms and the sequences of other studied species revealed a common pattern in the expanded repertoires, where IL-17 genes appeared repeatedly in tandem duplications in a single scaffold/chromosome. This observation was particularly notorious in the expanded repertoires of marine invertebrates such as *M. galloprovincialis*, *M. yessoensis*, *O. bimaculoides* and *S. purpuratus* and in the hemichordate *S. kowalevskii* (**Figure 5**).

The gene structure and genomic context were also analyzed (**Supplementary Table ST2**). The 33 IL-17 genes found in the mussel reference genome (LOLA) were located on 18 scaffolds, while the 8 receptor genes were located on 5 scaffolds. Gene structure was variable, being 2 exons the most repeated structure in IL-17 and receptors presenting a greater number of exons, up to 11. There was no clear synteny conservation between any mussel isoform and other studied species, which could had suggested a possible conserved ancestral IL-17 gene or isoform (**Supplementary Figure S4**). Synteny conservation was only maintained when studying phylogenetically close species that share homologous IL-17 genes, as mentioned previously with vertebrates (**Supplementary Figure S5**).

### IL-17 Modulation After a Bath Infection

**Figure 6:**
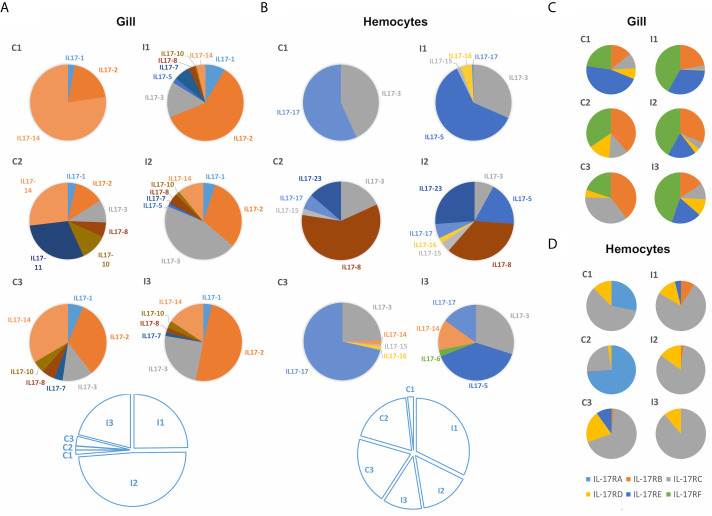
Expression of the different mussel IL-17 isoforms in the gill transcriptome against a waterborne bacterial infection **(A)** and in the hemocyte transcriptome against a bacterial infection injected into the muscle **(B)**. It is shown that the isoforms expressed in each transcriptome changed completely. Colored pie charts represent the diversity of expressed IL-17 isoforms in each individual mussel from the control (C1, C2, C3) and infected conditions (I1, I2, I3). The white pie charts below represent, for each experiment, the weight that the expression values of each of the upper graphs have (calculated by the sum of the expression values of every IL-17 form for each of the individual samples). Clear upregulation of IL-17 expression is seen in the gill transcriptome against infection but not in the hemocyte transcriptome. Expression of IL-17 receptors in the same gill **(C)** and hemocyte **(D)** transcriptomes. Regarding the IL-17 receptors, it can be seen that the expressed isoforms also varied between transcriptomes.

IL-17 expression retrieved from transcriptomic data of mussels subjected to bacterial infections differed among isoforms. Two transcriptomic experiments were analyzed: a bath or waterborne infection (24 h) sampled in gills (48) and a systemic infection (24h) sampled in hemocytes (49). As displayed in **Figures 6A, B**, the set of isoforms expressed was stimulus-specific and conditioned by the route of infection. There was a large diversity concerning which isoforms are expressed in each individual animal. Clusters such as IL17-3 or IL17-14 were among the most commonly expressed, while others were detected exclusively in the gill transcriptome samples (IL17-1, IL17-2 or IL17-10) or exclusively in hemocytes (IL17-17, IL17-15 or IL17-23). Some isoforms were even expressed only in a certain sample, i.e., not subjected to any stimulus or tissue (IL17-11, IL17-6). The bacterial waterborne infection increased the general expression levels of IL-17 forms in gills (**Figure 6A**). In hemocytes, changes in the expressed isoforms between individuals and between control and infected conditions were observed as well, but the differences in expression were not statistically relevant (**Figure 6B**). Receptors did not show the same diversity in terms of the different forms that are expressed in different individuals. However, it was noticeable that IL-17RA was not expressed in gills (**Figure 6C**) while IL-17RF was not expressed in hemocytes (**Figure 6D**).

**Figure 7:**
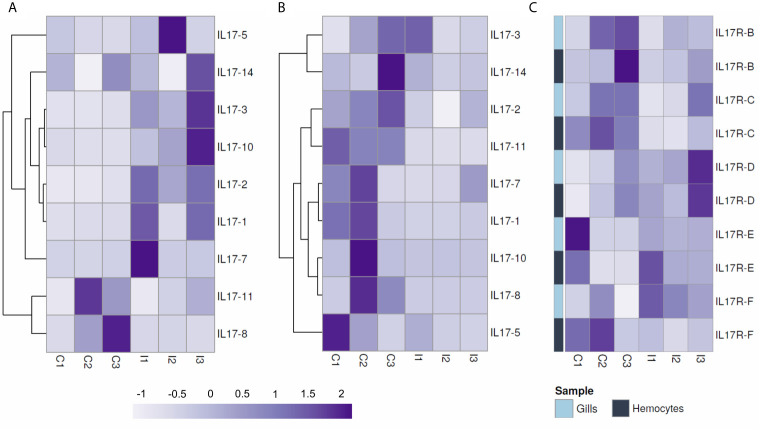
Normalized expression by qPCR of IL-17 isoforms expressed in the gill **(A)** and in the adductor muscle hemocytes of the same individual mussels **(B)** after a 24-hour waterborne infection with *Vibrio splendidus*, as well as the expression of the receptors **(C)**. In gills, upregulation of the expressed IL-17 isoforms occurred, whereas in hemocytes, there was downregulation. Receptors did not show the antagonistic regulation between hemocytes and gills seen with the IL-17 genes, but instead, the expression of each receptor was correlated in both gill and hemocyte samples.

To explore the modulation under a waterborne infection in more detail, the gene expression of the isoforms previously detected in the transcriptomic approach was studied by qRT-PCR. The expression of these IL-17 isoforms in gills and internal hemocytes differed completely. The general upregulation in gills of all expressed isoforms was confirmed, except for IL17-11 and IL17-8 (**Figure 7A**). Instead, in hemocytes sampled from the adductor muscle of the same individuals, IL-17 isoforms were generally downregulated (**Figure 7B**). The up-regulation in gills was significant for IL17-2 and IL17-3 while the down-regulation in hemocytes was significant for IL17-11 and IL17-2. Receptor isoform IL17R-D was up-regulated, while B and C were down-regulated (significant for IL17R-C in hemocytes). Expression in the isoforms E and F did not show any clear regulation trend. The expression of IL-17 receptors was analogous between gills and hemocytes for every expressed receptor, except for IL17R-F. The expression of isoforms IL17R-B, IL17R-C, IL17R-D and IL17R-E showed significant Spearman correlations between gills and hemocytes of 0.83, 0.77, 0.94 and 0.83 (**Figure 7C**).

The most highly expressed isoform (IL17-3) was studied by *in situ* hybridization in histological preparations of mussel gills. The results shown in **Supplementary Figure S6** may suggest that it is expressed not only in infiltrated hemocytes but also in epithelial gill cells.

## Discussion

The IL-17 proteins are key inflammatory cytokines encoded by a diverse, expanded gene family in mussels. As indicated previously, 6 IL-17 sequences and 3 receptors have already been described in mussels (35), but the advantages provided by genome sequencing and resequencing data (41), allowed us to reveal a whole new perspective on this gene family. The analysis of 16 mussel genomes revealed 379 unique IL-17 sequences. A similar level of diversity was observed for the receptors, with 96 unique variants. Once all of these sequences were clustered, there was conservation of almost every isoform among the 16 individual genomes. Therefore, this high variability seems to be different from the phenomenon of presence/absence variation (PAV) recently reported in *M. galloprovincialis* (41) and that characterizes other mussel immune genes. PAV events are associated with pangenomes, which are common among prokaryotes and certain plant species, wherein a core set of indispensable genes is shared among individuals, but there are large variations in genomic content affecting “dispensable” genes (one-third of all the protein-coding genes in mussels) present or absent among individual genomes (66, 67). This phenomenon may represent a breakthrough in the explanation of mussel survival success, since it reveals an adaptive strategy that endows this species with vast levels of variability in key gene families, mainly those related to immunity. In the case of IL-17, unique variants of each isoform were caused by the interindividual variability in specific positions, but every isoform was conserved among individuals. Concerning isoform definition, it was revealed by the phylogenetic analysis that isoforms IL17-3 and IL17-16 would be diverging from IL17-17, since the branch of the latter contains both isoforms. The distinction between these isoforms was maintained due to the homology criterion and the evidence of distinct transcriptomic expression of these isoforms.

Comparative genomics using the same clustering parameters with species from different phyla revealed similarly expanded IL-17 repertoires in other marine invertebrates, as well. This was observed mainly in marine bivalve mollusc species (*C. gigas* and *M. mizuhopecten*, in addition to the mussels *M. galloprovincialis* and *M. coruscus*), the cephalopod *O. bimaculoides* and the echinoderm *S. purpuratus*. Among molluscs, large IL-17 gene families have also been reported in more marine bivalves, such as *Pinctada fucata martensii* (33), while none of the gastropods studied possessed expanded repertoires. There was already previous evidence of IL-17 gene family expansions in octopi (32) and the purple sea urchin (30, 31), which we confirmed in this work.

In these marine invertebrate species, expansions are not restricted to IL-17, as these species are characterized to possess unusual large repertoires of other innate immune genes. This has been extensively reported in marine bivalves with C1q genes, pattern-recognition receptors, TLRs or TNF homologs (41, 68–73), in octopi with protocadherins, among others (32), and in the purple sea urchin with Sp185/333 proteins and TLRs, among other innate immune receptors (30, 74, 75). Expansion phenomena and diversification in innate immune gene families are usually associated with a higher degree of specificity, which endows certain invertebrates with more efficient immune responses. This has also been revealed on several occasions as a cause of the acquisition of innate memory capacity against certain pathogens (76).

The origin of these expanded IL-17 repertoires is not clear. Even if this proinflammatory cytokine is conserved across a wide range of species, the characteristics of the IL-17 family strongly differ among them, and there are no homologous forms that allow tracing throughout evolution. The fact that these expanded repertoires appeared in species from different phyla particularly enriched with highly variable immune genes points to an independent expansion in each lineage. This phenomenon would be supported by the observed tandem duplications of IL-17 genes in these species. The expanded repertoires are conserved within a specific lineage, and homologues are only found in the closest species, as observed between some Mytilidae and strongylocentroid echinoderm species (31). Nonmarine species and gastropods (nonfilter feeder molluscs) lacked these expanded repertoires, which could be related in a certain way to specific species in marine environments and filtering activity, both factors intrinsically linked to an increased exposure to pathogens. The lack of expanded repertoires happened as well in echinoderms as *Asterias rubens*, which is less closely related to *S. purpuratus*, supporting the discussed hypothesis regarding the independence of IL-17 expansions in specific species.

Nematoda had been proposed as the ancestral phylum of origin of IL-17 genes (28), but in the current work, we were able to identify IL-17 families in cnidarians, which emerged 600 million years ago. In the Pfam database, an IL-17 domain sequence was deposited from the poriferan *Amphimedon queenslandica* (ID: A0A1X7 VXB1_AMPQE/359-442), but this interleukin could not be identified in the sponge genome in our work or in previous approaches (28).

IL-17 is typically absent from some invertebrates, including insects and hemichordates (28); however, we found a rich IL-17 family in the hemichordate *S. kowalevskii.* Concerning insects, even if they are phylogenetically related to molluscs as protostomes, there are reports that marine molluscs and annelids are more closely related in terms of genomic organization, gene structure and functional content to some invertebrate deuterostomes, such as the sea urchin (77), which was also evidenced in this genomic study with IL-17 similarities. In the current work, we found three proteins in the *D. melanogaster* genome encoding the IL-17 domain. The three proteins had an amino acidic identity of 100% and differed only in length, therefore the more complete one was selected (NCBI ID: NP_001285550.1). These three proteins had been automatically annotated as prothoracicotropic hormones E, F and G but they share only 23.38% identity with the prothoracicotropic hormone from *D. melanogaster*, which did not contain the IL-17 domain (CAA66841.1). The putative *D. melanogaster* IL-17 should be further analyzed in order to verify its identity.

The study of IL-17 domains highlighted some evolutionary patterns. Invertebrate isoforms contain 6-8 conserved cysteine residues that conform to at least 3 disulphide bridges (28), while vertebrates and chordate invertebrates IL-17s are characterized by 2 bridges. These two missing cysteines have been replaced by serine residues. Motif analysis revealed a conserved motif CPW/SPW (with sporadic variations in the third amino acid) that appeared repeatedly in invertebrate and chordate sequences respectively.

Chordata species, both invertebrates and vertebrates, showed similar repertoires. Only in the Atlantic salmon did we find some additional genes clustered in a family of isoforms similar to humans and quite conserved in other teleosts (19, 21, 23). Expansion in certain marine invertebrates may not be necessary since specificity is guaranteed by their adaptive immune system. Considering that Chordata species contain a reduced number of IL-17 isoforms that are described to possess functional diversification, it seems reasonable that the same would happen in expanded repertoires as mussels.

Analyses of transcriptomic data indicated the importance of this sequence variability. Expressed isoforms varied among tissues, individuals and stimuli. There were also IL-17 isoforms that lacked the signal peptide, as reported previously in other invertebrate IL-17 repertoires (28). The reason behind this could be a misannotation that does not identify the exon coding for the signal peptide. However, this could be also related to putative functional diversification. In fact, variable exon-intron structures were reported for mussel IL-17 genes, ranging from 1 to 7 exons, although the 2 exon structure was the most repeated one. This is not rare, since invertebrate IL-17 genes are characterized by more variable exon-intron structures than vertebrates (28, 33).

Specific isoforms of the expanded *S. purpuratus* IL-17 repertoire were implicated in the gut epithelium immune response after bacterial infection in sea urchin larvae, activating the modulation of several other innate immunity genes. Another isoform was induced in adult immune cells, representing evidence of functional diversification of the repertoire (31). In mussels, an upregulation of IL-17 in the gills in response to a waterborne infection from the surrounding water has been reported (48). *In situ* hybridization with the most highly expressed isoform (IL17-3) suggested that these interleukins may be expressed in hemocytes and in the epithelial cells of gills. IL-17 isoforms responsive to waterborne bacterial infection could therefore exert specific immune functions in gills, which is in accordance with the role of IL-17 in other species, where they are also implicated in epithelial immunity (31, 78).

IL-17 in gill cells triggers the immune response when coupled to IL-17 receptors, triggering NF-kB signaling and activating antimicrobial peptide expression in molluscs, among other immune genes (37, 79–81). This suggests an ancient role of IL-17 in the mucosal immune responses of gills from marine animals. Indeed, the continuous filtering activity of bivalves could lead to repetitive contact with putative pathogens, for which they would need a strong arsenal of immune molecules and clear recognition of nonself-elements.

The current work indicates the importance of comparative immunology and genomics in studying elements of the innate immune response conserved throughout evolution. IL-17 gene families were described for the first time in several invertebrate species, and Cnidaria was found to be the most ancient diverged phylum. More diverse repertoires were observed in marine animals, which could be related to their environment rich in pathogens. Large expansions of IL-17 gene families were observed in certain phyla of marine invertebrates, revealing rich repertoires with functional diversification. Mussels are species characterized by presence/absence variation in key gene families, but even if they present high sequence variability, IL-17 isoforms are conserved among individuals. This could point to the great importance of these cytokines in the immune response of mussels and other marine invertebrates. We demonstrated the implications of these cytokines in mussel gills, including taking part in immune signaling after the recognition of an infection from the environment. The involvement of Il-17 in mucosal immune signaling seems to be conserved from ancestral lineages to humans.

## Data Availability Statement

The datasets presented in this study can be found in online repositories. The names of the repository/repositories and accession number(s) can be found in the article/**Supplementary Material**.

## Ethics Statement

The Mediterranean mussel, *M. galloprovincialis*, is not considered an endangered or protected species in any international species catalog, including the CITES list (www.cites.org), and it is not included in the list of species regulated by the EC Directive 2010/63/EU. Therefore, no specific authorization is required to work on mussel samples.

## Author Contributions

BN and AF conceived and designed the project. AS, MR-C, UR and AF performed the bioinformatic analyses. AS and MR-C performed the functional assays. AS wrote the manuscript. All authors contributed to the article and approved the submitted version.

## Funding

This research was funded by the Spanish AEI/EU-FEDER (RTI2018-095997-B-I00); the European Regional Development Fund (ERDF) Interreg V Spain—Portugal (0474_BLUEBIOLAB); and the Consellería de Economía, Emprego e Industria - GAIN, Xunta de Galicia, project IN607B 2019/01 and EU H2020, project VIVALDI (678589). SA was supported by a Spanish AEI/EU-FSE predoctoral contract PRE2019-090760. MR-C was supported by a Spanish AEI/EU-FEDER predoctoral contract BES-2016-076302.

## Conflict of Interest

The authors declare that the research was conducted in the absence of any commercial or financial relationships that could be considered as a conflict of interest.

## Publisher’s Note

All claims expressed in this article are solely those of the authors and do not necessarily represent those of their affiliated organizations, or those of the publisher, the editors and the reviewers. Any product that may be evaluated in this article, or claim that may be made by its manufacturer, is not guaranteed or endorsed by the publisher.
