# Supplementary material for: The Evolution and Diversity of Interleukin-17 Highlight an Expansion in Marine Invertebrates and Its Conserved Role in Mucosal Immunity
Source: Front Immunol. 2021 Jul 27;12:692997. doi: 10.3389/fimmu.2021.692997 (PMC8353272; doi:10.3389/fimmu.2021.692997)
Supplement: Supplementary file 1 [file DataSheet_1.zip › Supplementary Figures and Tables.pdf]

Supplementary Figures and Tables:

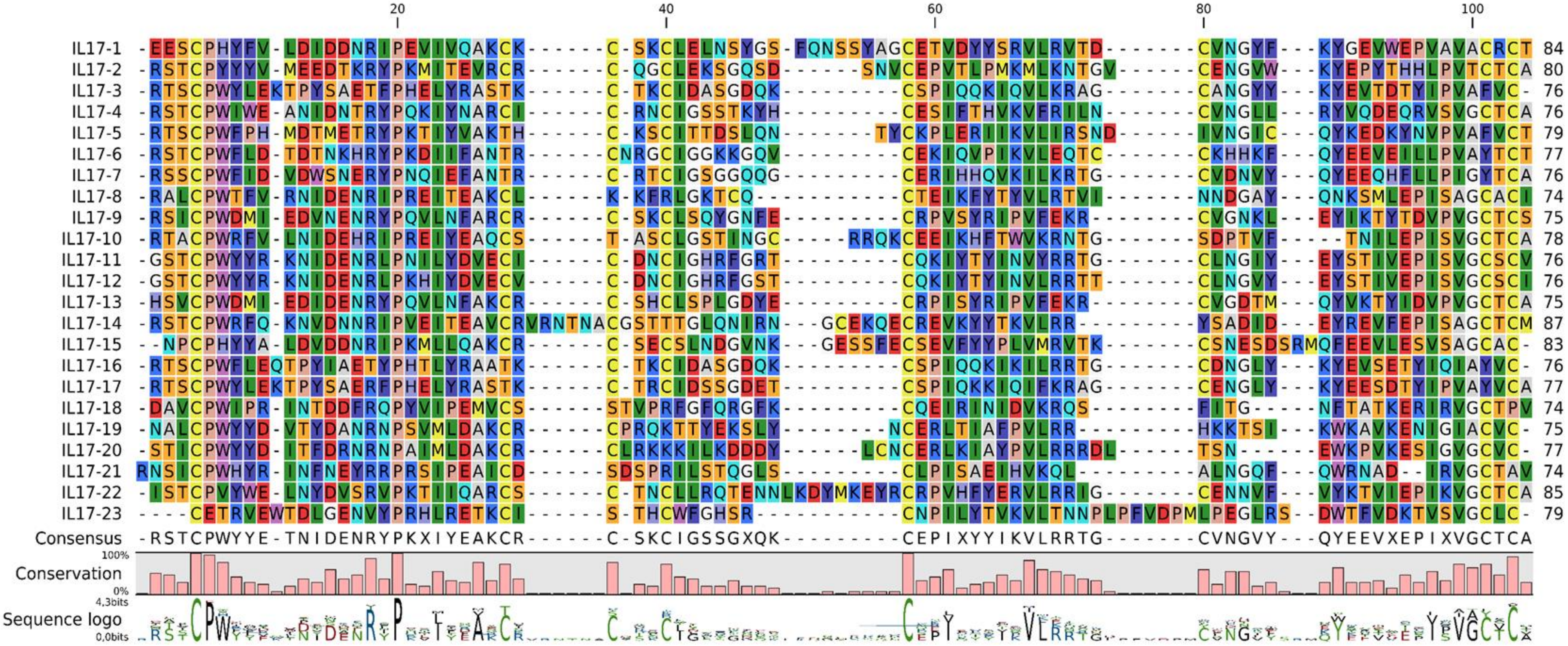

Supplementary Figure S1. Multiple sequence alignment with the IL17 domain of a representative sequence from each IL-17 mussel isoform. Conservation of each position is represented. The cysteine knot fold motif that distinguish the conserved structure of IL-17 is maintained in every sequence.

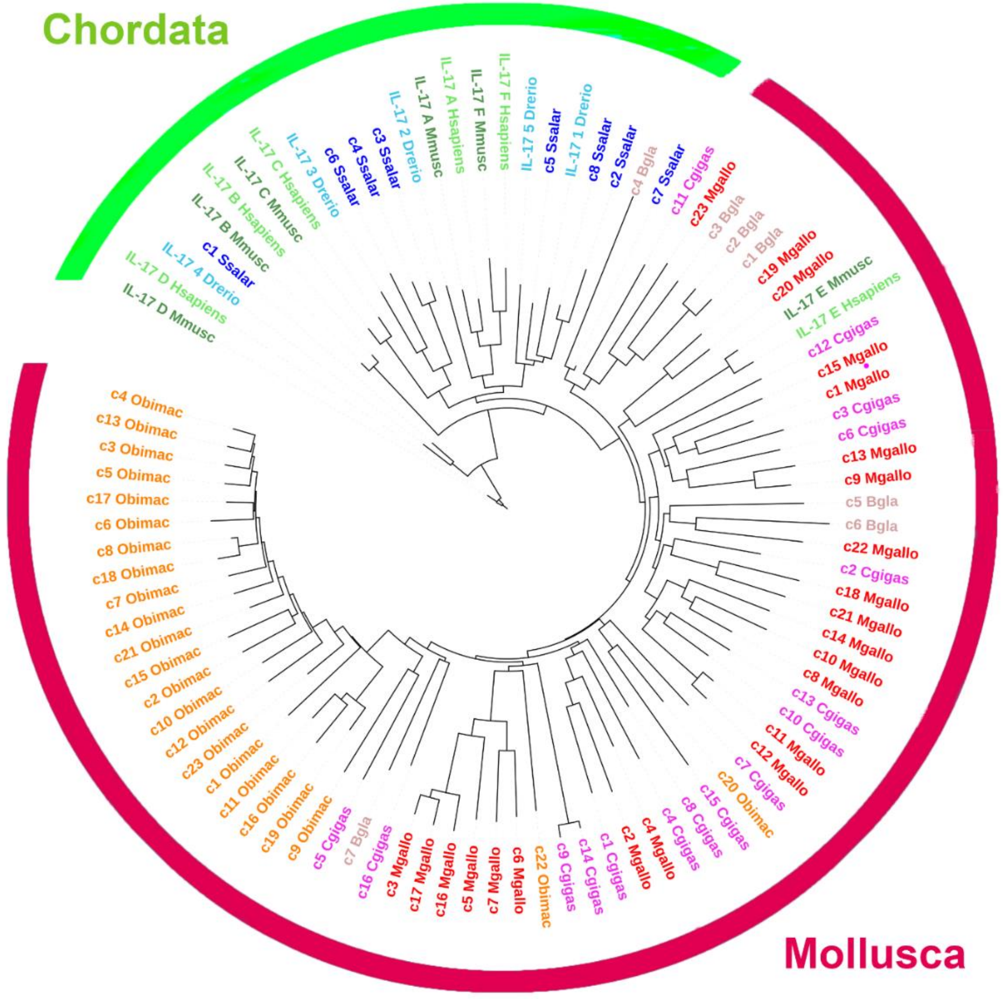

Supplementary Figure S2. Neighbor joining phylogenetic analysis of different selected species. Sequences from the repertoires of selected Chordata species (*Homo sapiens*, *Mus musculus*, *Danio rerio*, *Salmo salar*) and Mollusca species (*Mytilus galloprovincialis*, *Crassostrea gigas*, *Biomphalaria glabrata*, *Octopus bimaculoides*) were submitted to phylogenetic analysis. Results evidence the lack of homolog sequences between different phyla. The tree is clustered by phyla/species and not by isoform. Clustering by isoform can be only observed when working only with phylogenetically close IL-17 repertoires, with homolog genes.

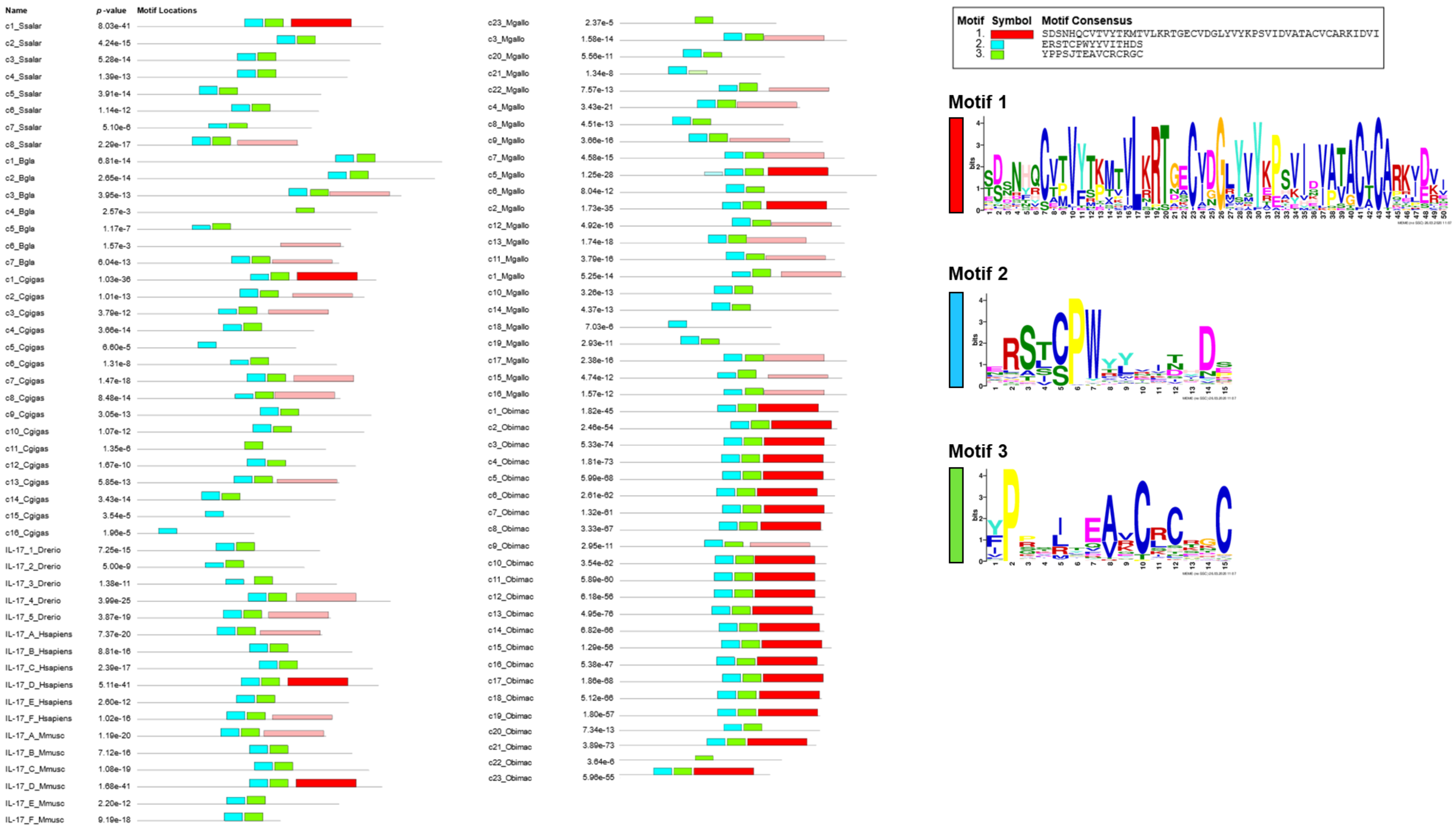

Supplementary Figure S3. Conserved motif analysis in representative sequences of IL-17 isoforms different selected species. Although the amino acid identity is low between sequences from different phyla, there is a great conservation at the motif level, relative to the functional IL-17 domain region. Conserved motif logos show the defining cysteine pattern and a conserved motif consisting in CPW in invertebrate sequences and SPW in chordate species.

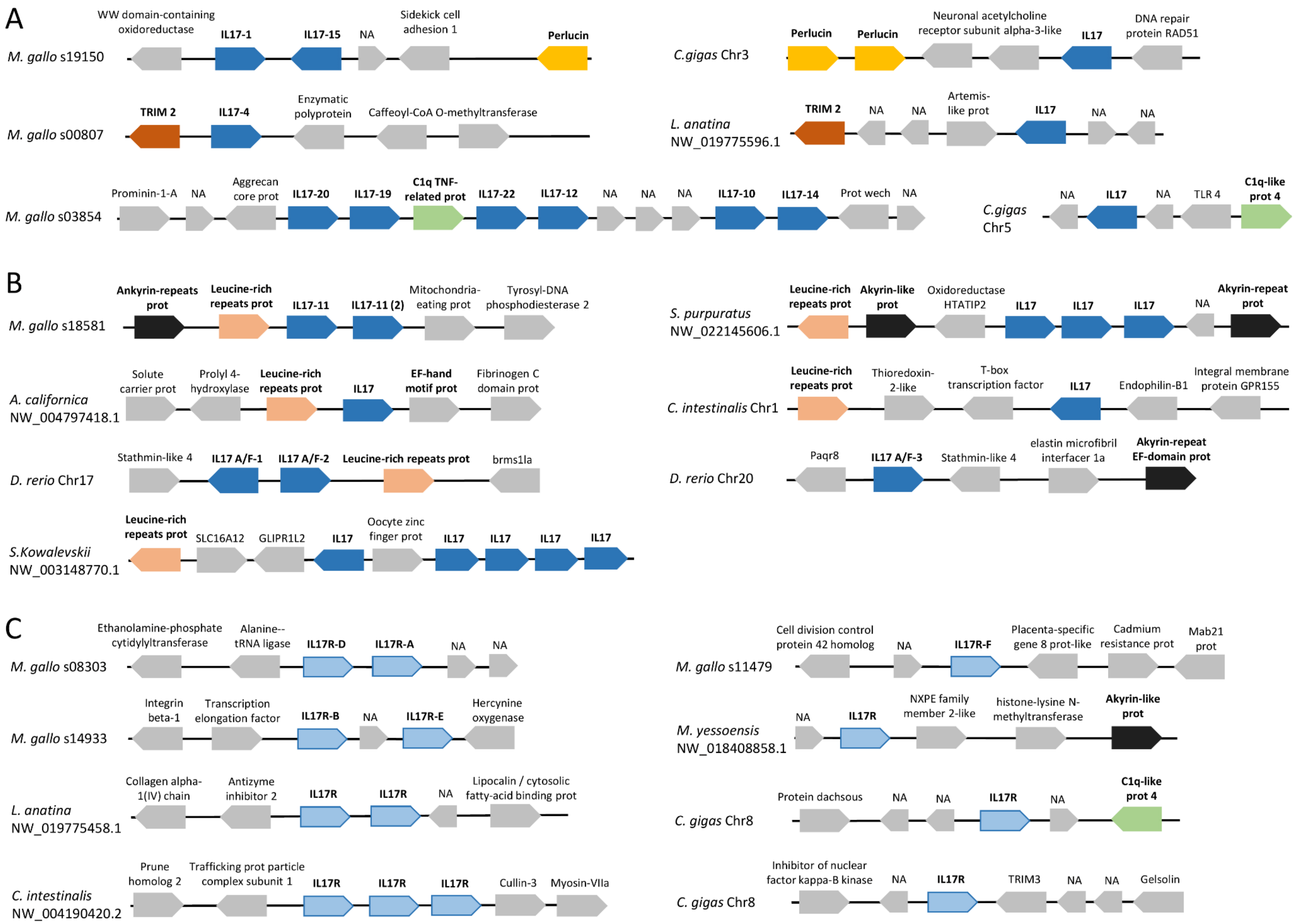

Supplementary Figure S4. Synteny conservation analyses between IL-17 genes throughout different phyla. The higher homologies found were presented, which evidences the absence of synteny conservation between the different IL-17 families. Genes were represented with their functional annotation to allow the visualization of putative conservations. Some interesting genes were found repeatedly, as Perlucin, TRIM-2, C1q-related proteins **(A)**, or more frequently Leucine-rich proteins and Ankyrin repeats proteins **(B)**. IL-17 receptor genes also did not show synteny conservation **(C)**.

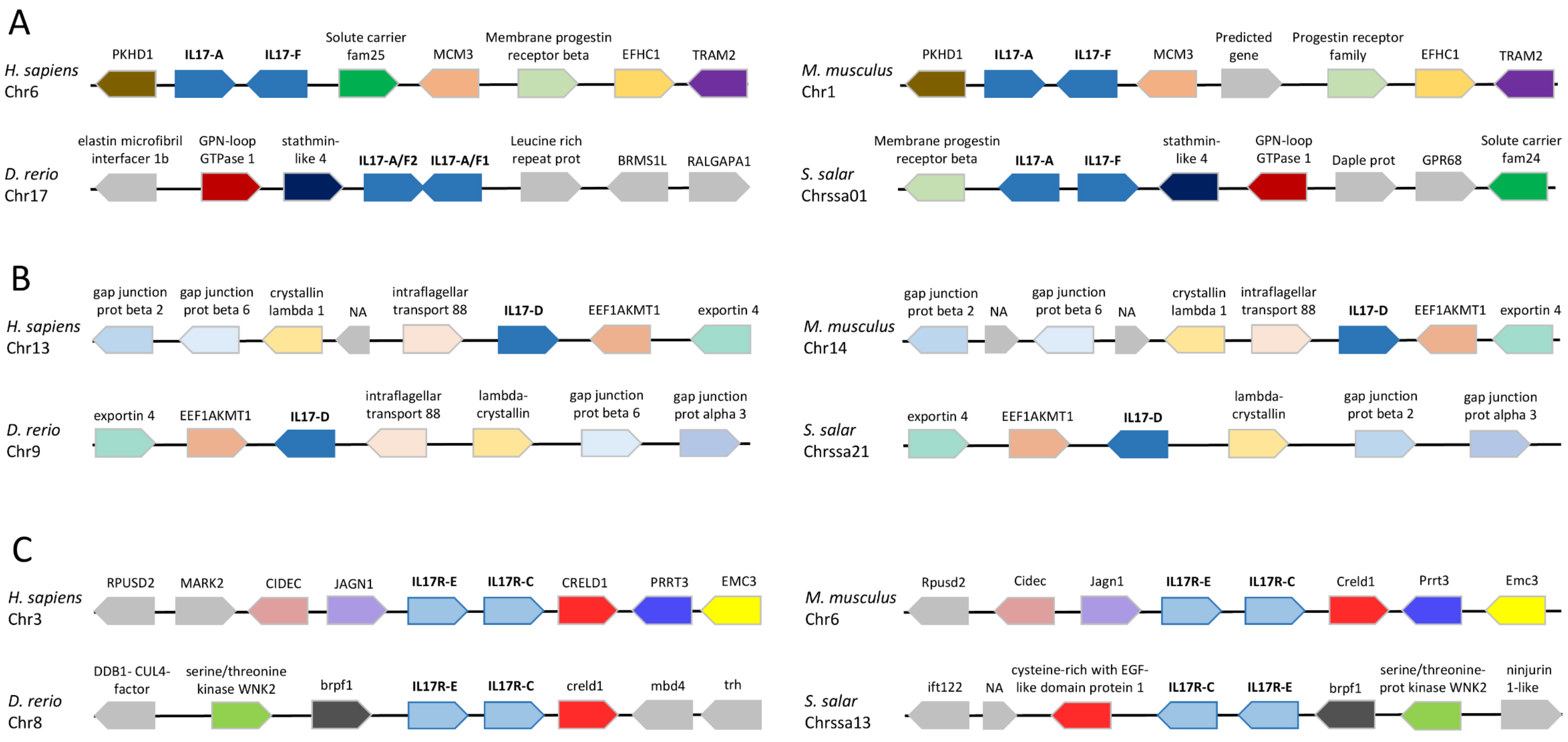

Supplementary Figure S5. IL-17 homolog genes among the repertoires of chordate vertebrate species did show synteny conservation. Instead of the absence of it between isoforms from different phyla, vertebrate species maintained synteny conservation as it is represented for IL17-A and IL17-F homologs **(A)**, IL17-D homologs **(B)** and IL17R-E and IL17R-C homologs **(C)**. Neighbor genes are represented with the official gene symbols in these species. It can be observed as well that in vertebrate species, some IL-17 genes appear together as well, but no tandem repetitions were observed as it happened in the expanded repertoires of marine invertebrates.

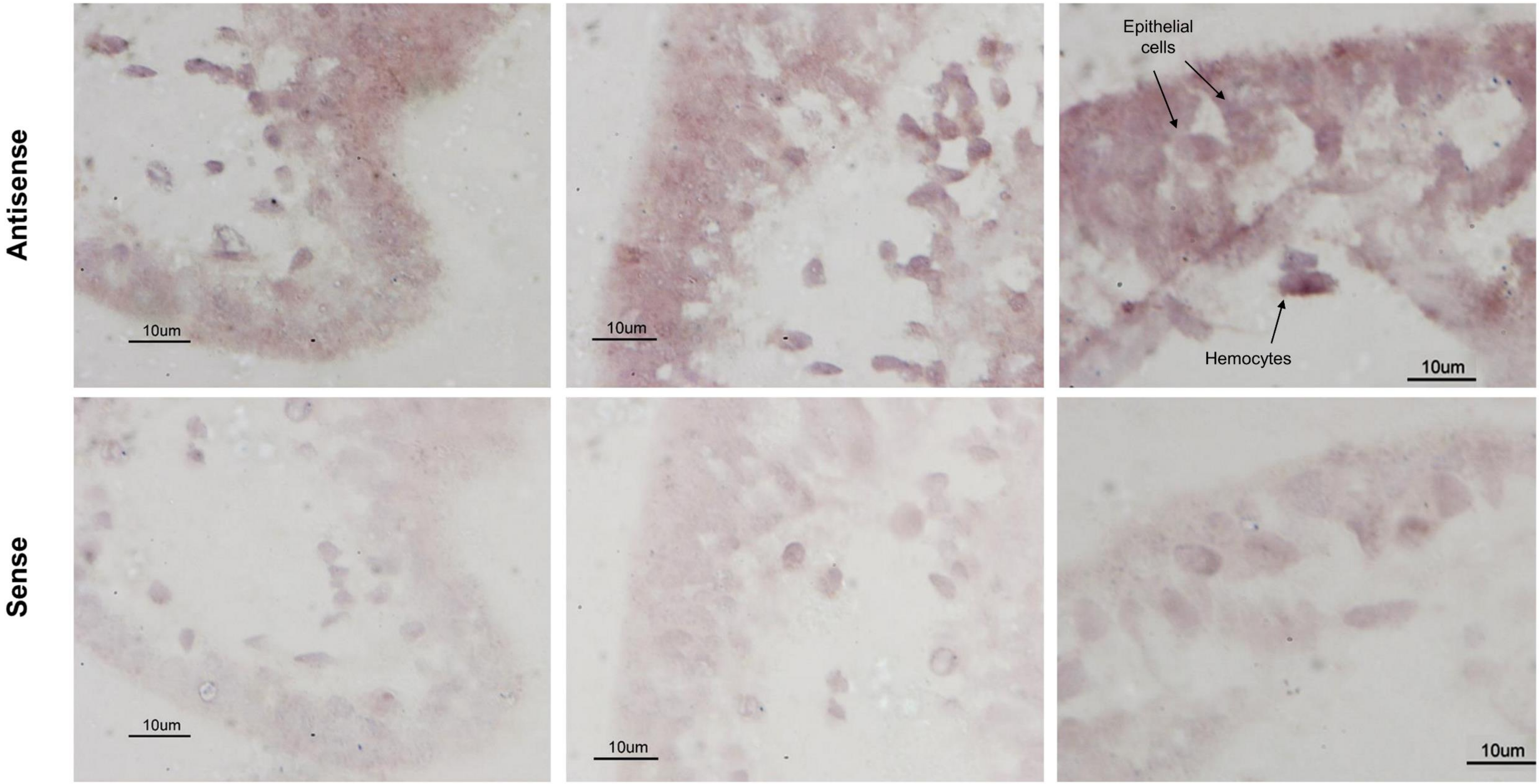

Supplementary Figure S6. IL17-3 expression at the mRNA level was detected by ISH in histologic preparations of mussel gills using specific antisense RNA probes. Control samples were hybridized with the sense probes. Replicate sections were used with sense and antisense probes. Alkaline phosphatase detection of hybridized antisense probes in comparison with the absence of colour reaction in the sense probe negative controls reveals expression of IL17-3 mRNA in mussel gills in both epithelial cells and infiltrated hemocytes.

Supplementary Table ST1. Primers and probes sequences used in the ISH and qPCR analyses.

Primers used for obtaining ISH specific RNA probes		
**IL17-3 F Sp6**	ACGATTTAGGTGACACTATAGAACGCTGTATGGCGTCTTCTCT	
**IL17-3 R T7**	AGTTAATACGACTCACTATAGGGATTTTTCACGGAAGGTTGGAC	
**qPCR primers**		**Efficiency**
**IL17-1 F**	TGGGCATCTTCCTTCTTTTG	-2.8438
**IL17-1 R**	TGAGACGGATACGAGCTGAG	
**IL17-2 F**	TACGCTGTCGTTGTCAAGGT	-3.2922
**IL17-2 R**	ACACCATTTTCGCAAACTCC	
**IL17-3 F**	CGCTGTATGGCGTCTTCTCT	-3.0403
**IL17-3 R**	TTTTTCACGGAAGGTTGGAC	
**IL17-5 F**	TTCGAGAAGGTGAATGACCA	-3.74
**IL17-5 R**	GCACATGCCCTATACCATCA	
**IL17-7 F**	CAACAGGGCTGTGAGAGGAT	-3.6593
**IL17-7 R**	ACGGGAACATCATCAGAAGG	
**IL17-8 F**	CTTGGACTTTTGTGCGGAAT	-3.9911
**IL17-8 R**	CCTGCCGAAATTGGTTCTAA	
**IL17-10 F**	GCGAGCTGTCTTGGGTCTAC	-3.3953
**IL17-10 R**	GCTTCGGACTGGAGATTGAA	
**IL17-11 F**	CATCCTTCATGGATATGGCA	-3.5256
**IL17-11 R**	TACTACCAACGGAGATCGGC	
**IL17-14 F**	GTACTGCGTCGTTATTCGGC	-2.966
**IL17-14 R**	ATGGCACATTGAATTACGGC	
**IL17R-B F**	TACCATGTCAATCGGAGCAA	-3.77
**IL17R-B R**	GGTTGTATTGGTTCGAGCGT	
**IL17R-C F**	AATTCCTTGCCCTCCTCACT	-3.4259
**IL17R-C R**	TCTTCGTGTTCCTCCAAACC	
**IL17R-D F**	ATGGATCAGCAGACGGTCAT	-3.1012
**IL17R-D R**	GGATCTGAGGGTCTTAGTGCC	
**IL17R-E F**	CAGGTTTCAACGAAGCAACA	-3.5102
**IL17R-E R**	TTTCTTGGTCAATGCTGTCG	
**IL17R-F F**	AACCTGTAGACGATGGCGAC	-3.3867
**IL17R-F R**	GGGAATAGCATCACCAGCAT	

Supplementary Table ST2. Genomic information about every IL-17 and IL-17R gene in the mussel reference genome.

IL17 Isoform	Scaffold	Strand	Start	End	Size	Exons	Introns	Genes upstream up to 100kb	Genes downstream up to 100kb
1	s19150	+	57693	58160	468	1	0	WW domain-containing oxidoreductase (40kb, -)	**Interleukin 17 isoform IL17-15 (20kb, -)**
15	s19150	-	77315	78739	1425	2	1	**Interleukin 17 isoform IL17-1 (20kb, +)**	NA (10kb, +), Sidekick cell adhesion molecule 1(20kb, -), Perlucin (70kb, -)
2	s08503	+	74410	76136	1727	2	1	N-methyl-L-tryptophan oxidase (20kb, +)	Vacuolar protein sorting-associated prot 72 (10kb, -)
2.2	s18819	+	97545	110094	12549	5	4	E3 ubiquitin-protein ligase rnf213-alpha (10kb, -), NA (10kb, +)	Vacuolar protein sorting-associated protein 72 homolog (10kb, -)
7	s00912	-	54015	57627	3613	2	1	Transposable element Tcb1 transposase (50kb, -), NA (20kb)	**Interleukin 17 isoform IL17-6 (10kb, +)**
6	s00912	+	61736	65453	3717	2	1	**Interleukin 17 isoform IL17-7 (10kb, -)**	**Interleukin 17 isoform IL17-3 (15kb, -)**
3	s00912	-	81336	82487	1151	2	1	**Interleukin 17 isoform IL17-6 (15kb, +)**	NA (10kb, +)
7.2	s19460	-	38559	41720	3162	2	1	NA (30kb, -), NA (20kb, +), NA (20kb, -)	**Interleukin 17 isoform 17-6 (6.2) (10kb, +)**
6.2	s19460	+	45809	47832	2024	2	1	**Interleukin 17 isoform IL17-7 (7.2) (10kb, -)**	**Interleukin 17 isoform IL17-3 (3.2) (20kb, -)**
3.2	s19460	-	65782	67169	1388	2	1	**Interleukin 17 isoform 17-6 (6.2) (20kb, +)**	NA (20kb, -)
4	s00807	+	37179	37631	453	1	0	Tripartite motif-containing protein TRIM 2 (30kb, -)	Enzymatic polyprotein (20kb, -), Caffeoyl-CoA O-methyltransferase (30kb, -) (60kb, +)
4.2	s11726	+	55664	56115	451			Retrovirus-related Pol polyprotein from transposon 412 (10kb, -)	Neurogenic locus notch homolog protein 1 (50kb, +)
5	s11170	+	627169	629544	2376	2	1	NA (90kb, -), C-type lectin domain family 7 member A (10kb, +)	
8	s04570	+	71540	84211	12672	7	6	NA (40kb, -), 2'-5'-oligoadenylate synthase 3 (30kb, +) (10kb, +)	Zinc metalloproteinase nas-36 (20kb, -)
13	s00443	-	185155	185718	564	1	0	NA (20kb, -), Small proline-rich protein 3 (15kb, -), NA (10kb, -)	**Interleukin 17 isoform IL17-9 (10kb, -)**
9	s00443	-	191805	192224	420	1	0	**Interleukin 17 isoform IL17-13 (10kb, -)**	
20	s03854	+	229973	230386	414	1	0	Prominin-1-A (60kb, +), NA (40kb, +), Aggrecan core protein (10kb, -)	**Interleukin 17 isoform IL17-19 (10kb, +)**
19	s03854	+	241593	241994	401	1	0	**Interleukin 17 isoform IL17-20 (10kb, +)**	Complement C1q TNF-related prot (5kb, +), **Interleukin 17 isoform IL17-22 (25kb, +)**
22	s03854	+	265989	266393	405			**Interleukin 17 isoform IL17-19 (25kb, +),** Complement C1q TNF-related prot (5kb, +)	**Interleukin 17 isoform IL17-12 (10kb, +)**
12	s03854	+	276125	277549	1425	2	1	**Interleukin 17 isoform IL17-22 (10kb, +)**	NA (5kb, +) (15kb, +) (20kb, +), **Interleukin 17 isoform IL17-10 (50kb, +)**
10	s03854	+	323680	324210	540			**Interleukin 17 isoform IL17-12 (50kb, +)**	**Interleukin 17 isoform IL17-14 (25kb, +)**
14	s03854	+	350092	352774	2683	2	1	**Interleukin 17 isoform IL17-10 (25kb, +)**	Protein wech (5kb, -), NA (30kb, +)
11	s18581	+	178618	179154	536			Ankyrin-repeats 3 prot (100kb, +), Leucine-rich repeat kinase 2 (15kb, +)	**Interleukin 17 isoform 17-11 (11.2) (10kb)**
11.2	s18581	+	187960	188499	450			**Interleukin 17 isoform 17-11 (11.1) (10kb)**	Mitochondria-eating prot (20kb, +), Tyrosyl-DNA phosphodiesterase 2 (70kb, -), Tyr-prot kinase (90kb, +)
16	s15501	-	140923	142624	1701	2	1	E3 ubiquitin-protein ligase HACE1 (70kb, +), NA (60kb, +), NA (40kb, +)	
17	s03945	+	12022	17664	5643	2	1		Myb/SANT-DNA-binding prot (30kb, -), fucosyltransferase (40kb, +), xylosyl-glucuronyltransf. (60kb, +)
18	s11501	+	197164	198319	1156	2	1	Baculoviral IAP repeat-containing protein 2 (90kb, -), Plexin-A4 (30kb, -), NA (20kb, -)	Chymotrypsin-like serine proteinase (40kb, -), Chymotrypsinogen B (50kb, -)
18.2	s18725	-	3489	3869	380	1	0		NA (5k, +), NA (8kb, -), **Interleukin 17 isoform IL17-18 (18.3) (10kb)**
18.3	s18725	-	13743	14123	380			**Interleukin 17 isoform IL17-18 (18.2) (10kb, -)**	NA (30kb, +) (35kb, +) (50kb, +)
21	s08023	-	12972	13323	351			NA (10kb, -)	**Interleukin 17 isoform IL17-21 (21.2) (8kb, -)**
21.2	s08023	-	20930	21280	351			**Interleukin 17 isoform IL17-21 (21) (8kb, -)**	**Interleukin 17 isoform IL17-21 (21.3) (6kb, -)**
21.3	s08023	-	27507	27860	354			**Interleukin 17 isoform IL17-21 (21.2) (6kb, -)**	Fatty acid-binding protein, heart (15kb, -), NA (50kb, -)
23	s11648	+	25242	25636	395			NA (20kb, +), NA (15kb, -)	NA (40kb, +), NA (50kb, -)
**Receptors**	**Scaffold**	**Strand**	**Start**	**End**	**Size**	**Exons**	**Introns**	**Genes upstream (10.000 pb)**	**Genes downstream (10.000 pb)**
A	s03747	-	334875	335949	9118	3	2	UPF0577 prot KIAA1324-like homolog (80kb, -), M-phase inducer phosphatase 3 (10kb, -)	**Interleukin 17 Receptor isoform IL17R-D (30kb, -)**
D	s03747	-	365629	403291	38096	11	10	**Interleukin 17 Receptor isoform IL17R-A (30kb, -)**	M-phase inducer phosphatase 3 (10kb, +), Pre-mRNA-splicing factor spp2 (40kb, +), NA (50kb, -)
D.2	s08303	+	46221	47398	1178	1	0	Ethanolamine-phosphate cytidylyltransferase (30kb, -), Alanine--tRNA ligase (5kb, -)	**Interleukin 17 Receptor isoform IL17R-A (A.2) (20kb, +)**
A.2	s08303	+	68708	79676	10968	6	5	**Interleukin 17 Receptor isoform IL17R-D (D.2) (20kb, +)**	NA (30kb, +), NA (40kb, +)
B	s14933	+	333175	352583	19409	11	10	Integrin beta-1-binding prot (40kb, -), Transcription elongation factor (15kb, +), NA (10kb, +)	NA (10kb, +), **Interleukin 17 Receptor isoform IL17R-E (30kb, +)**
E	s14933	+	379997	391333	11337	11	10	**Interleukin 17 Receptor isoform IL17R-B (30kb, +)**	Hercynine oxygenase (5kb, -), NA (30kb, -), NA (40kb, -)
C	s16435	+	38823	43348	4526	3	2	NA(50kb, -), NA (40kb, -)	-
F	s11479	+	55154	73446	18292	11	10	Cell division control protein 42 homolog (50kb, -), NA (10kb, +)	Placenta-specific prot (5kb, -), cadmium resistance prot (30kb, +), mab21 protein(60kb, -)
